# The phage gene *wmk* is a candidate for male killing by a bacterial endosymbiont

**DOI:** 10.1371/journal.ppat.1007936

**Published:** 2019-09-10

**Authors:** Jessamyn I. Perlmutter, Sarah R. Bordenstein, Robert L. Unckless, Daniel P. LePage, Jason A. Metcalf, Tom Hill, Julien Martinez, Francis M. Jiggins, Seth R. Bordenstein

**Affiliations:** 1 Department of Biological Sciences, Vanderbilt University, Nashville, Tennessee, United States of America; 2 Vanderbilt Microbiome Initiative, Vanderbilt University, Nashville, Tennessee, United States of America; 3 Department of Molecular Biosciences, University of Kansas, Lawrence, Kansas, United States of America; 4 Department of Pediatrics, University of Michigan, Ann Arbor, Michigan, United State of America; 5 Department of Genetics, University of Cambridge, Cambridge, United Kingdom; 6 Department of Pathology, Microbiology, and Immunology, Vanderbilt University, Nashville, Tennessee, United States of America; 7 Vanderbilt Institute for Infection, Immunology and Inflammation, Vanderbilt University, Nashville, Tennessee, United States of America; Stanford University, UNITED STATES

## Abstract

*Wolbachia* are the most widespread maternally-transmitted bacteria in the animal kingdom. Their global spread in arthropods and varied impacts on animal physiology, evolution, and vector control are in part due to parasitic drive systems that enhance the fitness of infected females, the transmitting sex of *Wolbachia*. Male killing is one common drive mechanism wherein the sons of infected females are selectively killed. Despite decades of research, the gene(s) underlying *Wolbachia*-induced male killing remain unknown. Here using comparative genomic, transgenic, and cytological approaches in fruit flies, we identify a candidate gene in the eukaryotic association module of *Wolbachia* prophage WO, termed *WO-mediated killing* (*wmk*), which transgenically causes male-specific lethality during early embryogenesis and cytological defects typical of the pathology of male killing. The discovery of *wmk* establishes new hypotheses for the potential role of phage genes in sex-specific lethality, including the control of arthropod pests and vectors.

## Author summary

Male killing is an adaptive trait for bacteria that are maternally transmitted through host populations. Such bacteria are common in arthropods and resultantly have significant impacts on host population size, mating strategy, and evolution. Moreover, male-killing bacteria are under recent scrutiny as a symbiotic strategy for arthropod pest and vector control. Despite decades of research, the microbial genetic basis of *Wolbachia*-induced male killing remains elusive. Here we demonstrate that a single gene from the eukaryotic association module in prophage WO of *Wolbachia* is a candidate for male killing as it recapitulates many aspects of the phenotype when transgenically expressed in fruit flies. This discovery represents a step forward in understanding new roles of phage WO genes in shaping arthropod hosts and may inform the potential use of male killing in worldwide pest and vector control strategies.

## Introduction

*Wolbachia* (order Rickettsiales) infect an estimated 40–52% of all arthropod species [1, 2] and 47% of filarial nematode species [3], making them the most widespread intracellular bacterial symbiont in animals. Concentrated in host testes and ovaries, *Wolbachia* primarily transmit cytoplasmically from mother to offspring [4, 5]. In arthropod reproductive tissues and embryos, *Wolbachia* deploy cunning manipulations to achieve a greater proportion of transmitting females in the host population. Collectively, these strategies are categorized as reproductive parasitism.

Male killing, or selective death of an infected female’s sons [6], is one such form of reproductive parasitism [7, 8]. It enhances the fitness of *Wolbachia*-infected females in three potential ways: (i) reducing brother-sister competition for limited resources [9], (ii) reducing inbreeding [10], and/or (iii) providing nutrients in cases where infected sisters cannibalize embryos of their dead brothers [10]. Male-killing *Wolbachia* are widespread in several major insect orders [11] and in pseudoscorpions [12]. In addition, male-killing *Spiroplasma* [13], *Rickettsia* [10], and *Arsenophonus* [14] occur in diverse hosts including flies [13], ladybugs [10], and wasps [14].

Male killing can have several significant impacts on host evolution [15–18]. For example, male death may lead to host extinction or reduce the effective population size of the host. As a consequence, theory specifies that fixation of deleterious alleles in host populations is more likely, and fixation of beneficial alleles is conversely less likely [19, 20]. Male killing can also impose strong selection on hosts to counter the sex ratios shifts and lethality [16]. Evolutionary outcomes include mate preference between uninfected males and females [11], a shift towards more mate-attracting behaviors by females or male mate choice [11], and suppression of the phenotype [16, 21–23].

As they manipulate arthropod reproduction to drive through host populations, *Wolbachia* are currently deployed in two vector control strategies: population suppression to reduce the population size of mosquitoes, and population replacement to transform mosquito populations that transmit pathogens to ones that cannot transmit pathogens [24, 25]. In these cases, mosquitoes are released with *Wolbachia* that cause cytoplasmic incompatibility (CI), in which offspring die in crosses between infected males and uninfected females. Notably, population genetic modeling demonstrates that male killing can be deployed in conjunction with population suppression techniques to speed up eradication or reduction of a target arthropod population and increase the likelihood of success [26]. However, the genetic basis of *Wolbachia* male-killing has remained a mystery for more than sixty years [27] and the causative gene of the *Spiroplasma* male-killing phenotype has only recently been reported [28]. Thus, potential vector and pest control applications of male killing have yet to be experimentally validated.

In this study, we sought to determine the genetic basis of the male-killing phenotype in *Wolbachia*. Our previous comparative genomic, transcriptomic, and proteomic analyses identified two prophage WO genes, *cifA* and *cifB*, that underpin the induction and rescue of CI by *w*Mel *Wolbachia* in *D*. *melanogaster* [29, 30]. *cifA* and *cifB* reside in the newly characterized eukaryotic association module of prophage WO that is enriched with many sequences predicted to have eukaryotic functions and homologies [29, 31, 32]. Building on this previous analysis, we pursued characterization of genes that may also be responsible for male killing. Notably, *Wolbachia* can be multipotent because some strains induce multiple reproductive parasitism phenotypes (e.g., CI and male killing) depending on the host background or environmental conditions [8, 22, 33, 34]. For example, the *w*Rec strain of *D*. *recens* causes CI in its native host, but it kills males when introgressed into the genetic background of its sister species, *D*. *subquinaria* [22]. Importantly, *w*Mel and *w*Rec share 99.7% nucleotide identity [35], which raises the hypothesis that the CI-inducing *w*Mel genome may also harbor male-killing genes.

A long-standing question is whether multipotency is due to pleiotropy of the same gene(s) expressing different reproductive parasitism phenotypes or alternatively if different genes underpin the various forms of reproductive parasitism. We previously assessed several reproductive parasitism gene candidates in *w*Mel *Wolbachia* for both male killing and CI, including *cifA* and *cifB*, and we ruled out their involvement in male killing [29]. However, other genes may still be involved. Although *w*Mel is not known to naturally cause male killing, it is of interest because it is the native strain of the only host that is genetically tractable and is closely-related to a natural male killer, making it a useful system to test gene candidates for the phenotype.

There are several expectations for a putative *Wolbachia* male-killing gene. First, we expect transgenic expression will recapitulate the embryonic cytological defects typically induced by male killing [36]. Second, native expression of the candidate gene will occur by the time male death naturally occurs in a given host [22, 36]. Third, a male-killing gene would be shared across male-killing strains in *Wolbachia* but not necessarily absent from strains unknown to cause male killing. In other words, the gene may be more common than the phenotype because hosts frequently develop resistance to male killing, presumably due to the strong evolutionary pressure to avoid extinction [16, 21, 22, 37, 38]. As previously mentioned, *Wolbachia* can induce either male killing or CI in different hosts or rearing conditions [8, 21, 22, 33], which may be related to resistance in some hosts. Fourth, if there is a single gene that causes male killing in most or all cases, then the gene may rapidly evolve due to natural selection in diverse host backgrounds that suppress male killing. Here, based on genomic analyses, transgenic expression, and cytological characterizations in *Drosophila melanogaster* infected or uninfected by *w*Mel *Wolbachia*, we report the discovery of a gene in the eukaryotic association module of prophage WO that is a candidate for male killing.

## Results

### Genomic analysis of male-killing gene candidates

To generate a shortlist of male-killing gene candidates, we used the following criteria and assumptions: (i) universal presence in the genomes of male-killing strains *w*Bif from *D*. *bifasciata* [27], *w*Inn from *D*. *innubila* [7], *w*Bor from *D*. *borealis* [39], and *w*Rec from *D*. *recens* [22]; (ii) genomic location in prophage WO because parasitic *Wolbachia* all have intact or remnant prophage WO regions with eukaryotic association module genes [32]; notably, the two previous parasitism genes, *cifA* and *cifB*, are both in this module of prophage WO, making it likely that other parasitism genes share a similar origin; (iii) exclusion of highly repetitive elements, including insertion sequence elements, reverse transcriptases of group II intron origin, and large serine recombinases that likely facilitate phage WO lysogeny; and (iv) exclusion of disrupted genes (e.g., early stop codons) in one or more strains (S1 Table for list of excluded genes).

Table 1 shows seven candidate genes that fit these criteria. One of these genes, *cifA*, was previously evaluated by transgenic expression [29], and it did not exhibit a biased sex ratio. Others include a predicted ankyrin repeat (WD0550), two Rpn genes (recombination-promoting nucleases WD0297, WD0627), Phospholipase D (WD1243), and a hypothetical protein (WD0628). The remaining gene, WD0626, was identified in the previous multi-omic analysis that uncovered the *cif* genes [29]. This candidate gene, hereafter denoted *wmk* for *WO-mediated killing*, is a putative transcriptional regulator in prophage WOMelB that is predicted to encode two helix-turn-helix (HTH), XRE family DNA-binding domains (NCBI conserved domains E = 5.9 x 10^−11^_,_ E = 6.5 x 10^−10^). *wmk* in *w*Mel has a single amino acid difference relative to its homolog in *w*Rec. Due to the association of *wmk* with two different candidate gene analyses for reproductive parasitism and preliminary observations that transgenic expression associated with a sex ratio bias, we further assessed it as a putative male killing gene.

**Table 1:** Comparative genomic analysis of male-killing gene candidates. After applying all criteria in the genomic analysis, seven candidates for male killing were identified. All seven gene candidates are listed with their functional annotation and locus tags from both *w*Mel and the closely related *w*Rec strain. BLASTP results of the homologs are also shown with the percent coverage, E-value, pairwise identity, and number of nucleotides for each strain. For inclusion and exclusion criteria, see S1 Table. WD0626 from *w*Mel is the gene hereafter denoted *WO-mediated killing* or *wmk*.

Annotation	*w*Mel Locus Tag	*w*Rec Locus Tag	Ref-Seq Coverage	E-Value	Pairwise % Identity	*w*Rec	*w*Mel
**Ankyrin Repeat**	WD0550	wRec0541	100%	0	95%	789	990
**Transcriptional Regulator**	WD0626	wRec0560	100%	0	99%	912	912
**Rpn (Recombination-Promoting Nuclease)**	WD0627	wRec0561	100%	0	99%	897	897
**Hypothetical Protein**	WD0628	wRec0562	100%	0	100%	540	540
**CifA (CI Component)**	WD0631	wRec0566	100%	0	99%	1425	1425
**Rpn (Recombination-Promoting Nuclease)**	WD0296	wRec0561	81%	0	88%	897	912
**Phospholipase D**	WD1243	wRec1232	100%	0	99%	531	531

### The *wmk* gene is common and found in all sequenced male-killing genomes

**Fig 1:**
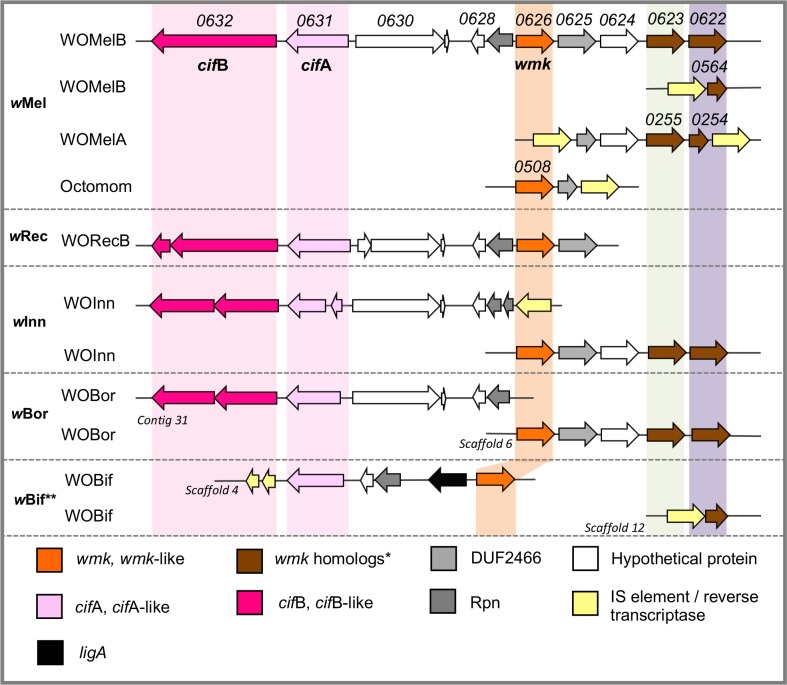
Comparative genomics of *wmk* and its homologs in *w*Mel and male-killing strains. Prophage WO gene regions containing *wmk*, *wmk*-like homologs, and CI genes *cif*A and *cif*B are listed by *Wolbachia* strain in bold and then prophage. At least one *wmk* homolog is associated with each *Wolbachia*-induced male killing strain. Genes pointing in the same direction are on the same DNA strand. The distance between *wmk* and *cif*A is approximately 5 kb. Shading highlights homologs in each strain. (*) *wmk* homologs are annotated as transcriptional regulators in the *Wolbachia* reference genomes and encode helix-turn-helix XRE domains (S4 Table). (**) While *w*Bif reportedly induces weak CI after temperature treatment [8], the assembled genome does not contain *cifB*.

Phylogenetic analyses indicate that *wmk* homologs are common in phage WO-containing *Wolbachia* including the above-mentioned male-killing strains (S1 Fig), *w*Bol from *Hypolimnas bolina* butterflies (causes CI when male killing is suppressed) [16, 21], and *w*CauB from *Cadra cautella* moths (causes male killing in non-native host) [33], along with many strains not known to cause male killing (S1A Fig). *wmk* is in the eukaryotic association module of prophage WOMelB, resides just a few genes away from the *cif* genes, and exists in multiple divergent copies in some strains (S1B Fig and Fig 1) [32]. Phylogenetic analyses indicate that *wmk* sequence relationships do not cluster into typical *Wolbachia* supergroups (S1A Fig), specifying independent evolution relative to the core *Wolbachia* genome. This finding is similar to that of other prophage WO genes including *cifA*, *cifB*, and the baseplate assembly gene, *gpW* [29]. It is attributable to the high rates of horizontal phage WO transfer between *Wolbachia* coinfections [40]. Similar to *cifA* and *cifB* [41], *wmk* homologs are notably disrupted in the parthenogenesis-inducing *Wolbachia* strains *w*Uni from *Muscidifurax uniraptor* wasps, *w*Tpre from *Trichogramma pretiosum* wasps, and *w*Fol from *Folsomia candida* springtails. The gene is also absent in the male-killing MSRO strain of *Spiroplasma poulsonii*, which contains the recently reported male-killing gene, Spaid [28]. Spaid has OTU deubiquitinase and ankyrin repeat domains and lacks direct homologs in *Wolbachia* [28], indicating separate evolutionary origins of Spaid and *wmk*. In addition, genomic analyses suggest the full version of *wmk* in phage WO potentially originated from a fusion or duplication event with gene(s) in the non-prophage region of the *Wolbachia* chromosome. Indeed, homologs of the N-terminal XRE-family HTH domain occur in distantly related nematode *Wolbachia* strains (*w*Wb, *w*Bm, *w*Ppe) and the sister genera *Ehrlichia* (S2 Table) that all lack prophage WO.

### Transgenic expression of *wmk* causes a female-biased sex ratio

To evaluate the function of *wmk*, we generated transgenic *D*. *melanogaster* flies that express codon-optimized *wmk* with the Gal4-UAS expression system because genetic editing of *Wolbachia* is not currently possible. We evaluated three other transgenes in a similar manner: WD0625 in prophage WO that encodes a putative MPN/Mov34/PAD-1 metalloprotease domain (DUF2466, NCBI conserved domain E = 3.85 x 10^−41^) because it is adjacent to *wmk* and may in theory be cotranscribed with *wmk*, WD0508 in the prophage WO-associated Octomom region that is another predicted transcription regulator with two XRE-family HTH DNA-binding domains (NCBI conserved domains E = 1.70 x 10^−9^, E = 1.99 x 10^−11^, a homolog of *wmk*), and WD0034, a non-phage, hypothetical protein-coding gene that is hereafter labeled ‘control gene’ and shares a transgenic insertion site with *wmk*. These three genes do not recapitulate CI [29]. In the experiments below, all transgenes were expressed in heterozygous flies under the control of an *Act5c*-Gal4 driver, which leads to ubiquitous transgene expression beginning with zygotic transcription ~2h after egg deposition (AED). Genetic crossing schemes are described in the methods.

**Fig 2:**
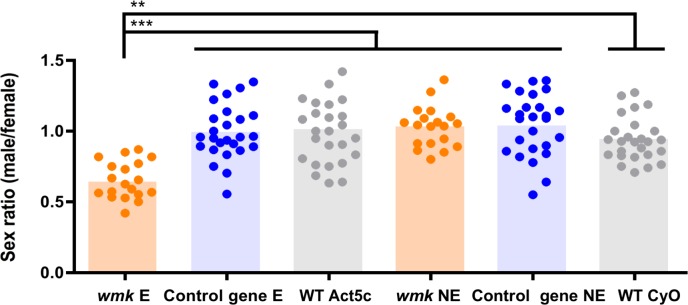
Transgenic expression of *wmk* causes a female-biased sex ratio. Each sample point represents the adult offspring produced by a replicate family of ten mothers and two fathers (average offspring number per data point is 90). Bars represent the average sex ratio. Control gene flies have the *Wolbachia* transgene WD0034. WT is the BSC8622 strain. E = expressing, NE = non-expressing, *Act5c* has an *Act5c*-Gal4 gene, CyO has the CyO chromosome. *wmk*-expressing flies have a significantly female-biased sex ratio against all other genotypes. This experiment has been done four times. Statistics are based on a Kruskal-Wallis one-way ANOVA followed by Dunn’s correction. **p<0.01, *** p<0.001. Orange dots represent *wmk*, blue dots represent the control gene, and gray dots represent the WT strain.

To assess if *wmk* causes sex-specific lethality, we first quantified adult sex ratios in gene-expressing (*Act5c*-Gal4; UAS-*wmk*) flies using a ubiquitously-expressing actin (*Act5c*) driver. *wmk* transgene expression results in a significant reduction in the average male:female sex ratio (number of males / number of females) to 0.65, or a 35% reduction in gene-expressing males (Fig 2). The sex ratio is approximately 1 in wild type flies and in transgenic flies that either do not express *wmk* (CyO; UAS-*wmk*) or express a control gene (Fig 2). All sex ratios represent a normal range of variance observed in previous experiments [28, 29, 42–44]. For example, natural male-killing *Wolbachia* strains cause variable offspring sex ratios that range from 0.5 to 0 (all females) in *D*. *innubila* [45, 46], and 0.2 to 0 in *D*. *subquinaria* [22], although most cases are all female. For the three other prophage WO genes, transgenic expression in uninfected flies does not significantly change sex ratios (S2A–S2C Fig), indicating the *wmk*-induced phenotype is not due to a generalized, transgenic artifact. Further, we explored whether another gene could be additionally involved. We tested dual expression of *wmk* and WD0625, as they are adjacent and could potentially function together. Dual expression does not change the degree of male death (S2A–S2C Fig), demonstrating it is not involved in the phenotype. In addition, ovarian transgene expression of *wmk* by the maternal triple driver (MTD) that loads product into developing oocytes [47] did not result in a biased sex ratio (S2D Fig) despite confirmed expression (S2E Fig). The lack of phenotype under the MTD driver is likely due to insufficient transcript levels in the embryo as MTD is a germline-specific driver expressed in mothers before eggs are laid, whereas *Act5c* is ubiquitously expressed by the embryo itself. However, transgenic expression of *wmk* via the *armadillo* driver, which expresses genes ubiquitously beginning in embryogenesis, yields sex ratios that are similar to that of the *Act5c* driver (S3A Fig), despite an order of magnitude reduction in expression level (S3B Fig). These findings indicate that expression at *Act5c* levels is not necessary to induce the phenotype, and zygotic transcription (~2 h AED) of *wmk* is required for the sex ratio effect. Thus, investigations so far have not revealed conditions that might alter the proportion of male death. Notably, this finding parallels the timing of embryonic mortality during early zygotic transcription in the *D*. *melanogaster* male-killer, *Spiroplasma poulsonii*, although it differs in that maternal expression does not recapitulate the *wmk* phenotype, while some aspects of the *Spiroplasma* phenotype can be recapitulated with maternal expression [28].

The *wmk*-induced change in sex ratio is also not consistent with other types of reproductive parasitism for several reasons. First, CI is not known to have a sex ratio bias except in haplodiploid species [29]. Second, the male lethality phenotype and transgene expression begin long after hallmark CI defects such as delayed histone deposition in fertilized embryos [48]. Third, an infected maternal background does not rescue the *wmk* phenotype, as would be expected if the phenotype were linked to CI (S4A Fig). Fourth, neither *wmk* expression nor dual expression of *wmk* and WD0625, a putative partner gene due to its adjacent location, causes or rescues CI when expressed with the *nanos*-Gal4 driver used in CI experiments for germline-specific expression [29] (S4B and S4C Fig). Fifth, the bias in sex ratio cannot result from genetic males developing as females (feminization) because *wmk* expression does not increase the absolute number of females compared to controls (S4D Fig). Finally, parthenogenesis (virgin females produce all female offspring) cannot explain the male lethality phenotype because transgenic expression occurs with a paternal chromosome present.

### Transgenic expression of *wmk* recapitulates embryonic death and cytological defects

**Fig 3:**
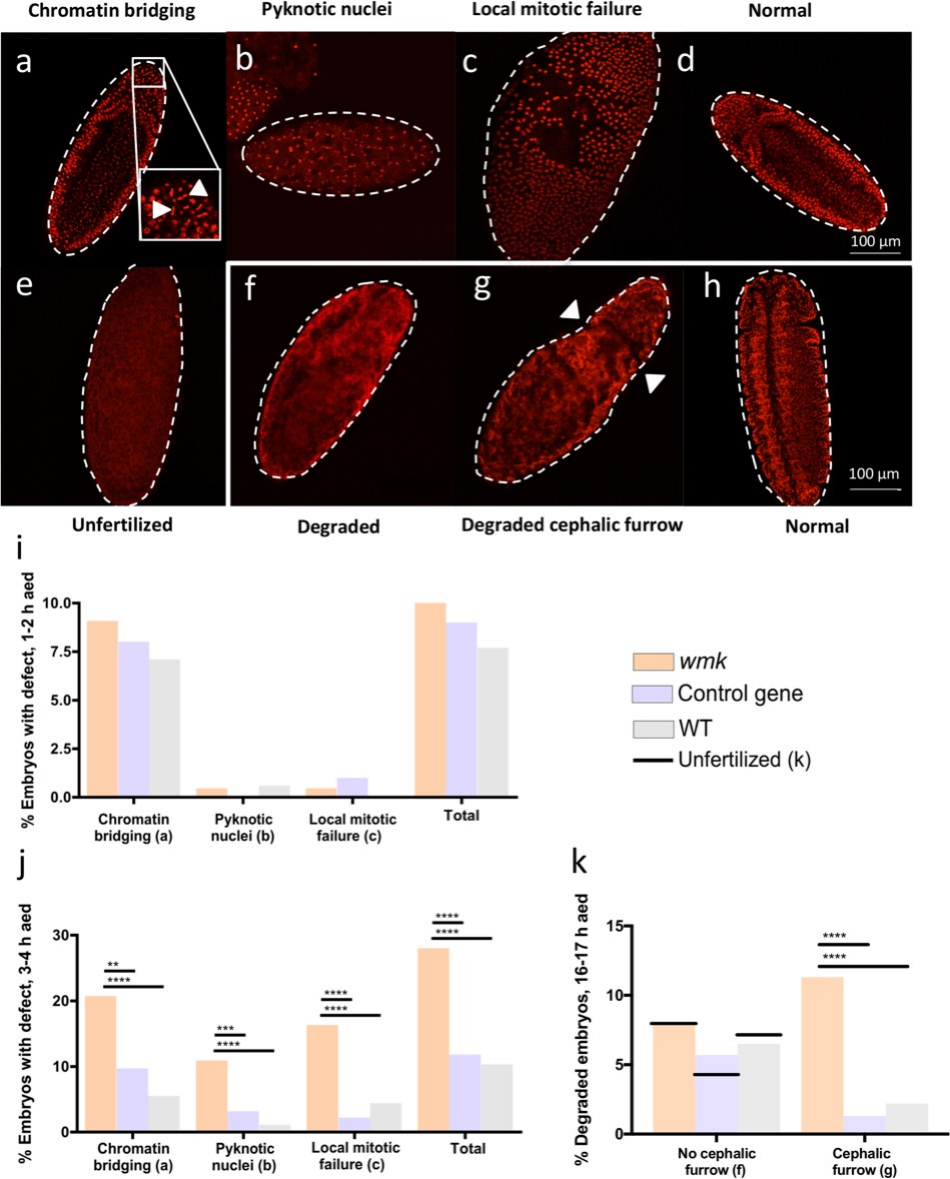
Transgenic expression of *wmk* causes cytological defects in early embryogenesis. Data are from pooled embryos (both sexes, expressing and non-expressing) with either *wmk*, the control gene, or an uninfected wild type (WT) background (see methods). (A-C) Defective *wmk* embryos fixed 3–4 h after egg deposition (AED) exhibit either chromatin bridging (arrowheads), pyknotic nuclei, or local mitotic failure leading to gaps in the distribution of nuclei, respectively. (B) Image has been brightened for visibility. (D) Image of a normal control gene embryo fixed 3–4 h AED. (E) Image of unfertilized embryo fixed approximately 3–4 h AED. (F) Image of degraded *wmk* embryo fixed 16–17 h AED with no distinct nuclei and no visible segmentation. (G) Image of a degraded *wmk* embryo fixed 16–17 h AED with no distinct nuclei, but the cephalic furrow is (indicated by arrowheads). (F) and (G) are brightened in order to see their differences. (H) Image of normal control gene embryo fixed 16–17 h AED. (I) Graph quantitating the percentage of embryos exhibiting DNA defects that were fixed 1–2 h AED. N = 220 for the *wmk* cross, N = 200 for the control gene cross, and N = 169 for the WT cross. Total refers to the total percentage of embryos with one or more of the three defects (embryos can have more than one, as in (A)). All differences within each defect category were not statistically significant. (J) Graph of the percentage of embryos exhibiting DNA defects that were fixed 3–4 h AED for *wmk*, control gene, and WT crosses. N = 276 for the *wmk* cross, N = 273 for the WT cross, and N = 279 for the control transgene cross. (K) Graph of the percentage of degraded embryos fixed 16–17 h AED in the *wmk*, control gene, and WT crosses. N = 327 for the *wmk* cross, N = 315 for the control transgene cross, and N = 231 for the WT cross. The percent of unfertilized eggs is the expected percent given the observed rate of unfertilized sibling eggs fixed 3–4 h AED (*wmk*, 8%, N = 324; control gene, 4.5%, N = 202; WT, 7%, N = 217). Statistics for (I), (J), and (K) were performed with a Chi-square test comparing the three genotypes within each defect category. These experiments have been performed once. The white border around (F, G, & H) indicates embryos fixed 16–17 h AED, while the rest (A-E) are embryos fixed 3–4 h AED. All images were taken at 20X zoom, except the inset image in (A) that is a zoomed in image of the same region. ** p<0.01, *** p<0.001, ****p<0.0001.

*Wolbachia*–induced male killing occurs either during embryogenesis or larval development in *Drosophila* [22, 36, 45]. Embryonic cytological defects associated with *Wolbachia* male killing begin largely at the time of host embryonic cellularization (~2.5 h after egg deposition, AED) and span abnormal nuclei distribution, chromatin bridging, and pyknosis in male embryos of *D*. *bifasciata* [36]. To determine if *wmk* transgene expression in *D*. *melanogaster* recapitulates the nature and timing of the defects, we stained DNA with propidium iodide in wild type (WT) embryos and in embryos expressing either *wmk* or the control transgene. We then monitored the defects in embryos (only half of the embryos are expected to express the transgene, see methods). Several different defects were observed (Fig 3A–3D). In embryos fixed 1–2 h AED, there was no significant difference in cytological defects of *wmk*-associated offspring compared to controls (Fig 3I). However, in embryos fixed 3–4 h AED, cytological defects were enriched in *wmk*-associated embryos (28%) relative to control gene (11.8%) and wild type embryos (10.3%) (Fig 3J). Since significantly more defects occur in embryos fixed 3–4 h AED but not in those fixed 1–2 h AED, the male lethal defects could commence between 2–4 h AED. These results also indicate that cytological defects specifically occur soon after zygotic transcription of *wmk*, as only a zygotic driver, not a maternal egg loader, is able to induce the phenotype.

In *w*Bif-infected *D*. *bifasciata*, male embryos 15–20 h AED have several large defects including incompletely formed regions and lack of differentiation or segmentation [36]. To determine if the defects in early *wmk*-expressing embryos result in similar abnormalities later in development, we fixed sibling embryos 16–17 h AED. We discovered and assessed degraded embryos (embryos with cloudy staining from degraded DNA and lack of distinct nuclei) in *wmk*-associated offspring compared to controls. One category of degraded embryos had no visible cephalic furrow or segmentation similar to unfertilized eggs (Fig 3E and 3F). These embryos occurred equally across all treatment groups at a low percentage similar to that of unfertilized eggs (Fig 3E and 3K). This category likely represents decomposing, unfertilized eggs. A second degraded form exhibited a cephalic furrow that demarcates the head from the thorax (Fig 3G), but it lacked other normally visible segmentation (Fig 3H), similar to the lack of segmentation in infected embryos. There were approximately 10-fold more degraded embryos with a cephalic furrow in the *wmk* cross versus controls (Fig 3K). This finding suggests the timing of death is soon after the commencement of the cephalic furrow formation, which occurs at approximately 3 h AED. As noted above, it is also approximately the time point when cytological defects are first observed (Fig 3J). The furrow formation is largely complete by 4 h AED, and it is visible in the degraded embryos, suggesting most embryos reach this developmental time point before death. Though this furrow phenotype is not described in natural contexts, the literature demonstrates that there are highly defective areas in embryos later in development [36]. The furrow phenotype likely occurs in transgenic individuals because of consistent, strong expression of a transgene rather than natural expression levels that may vary in individuals due to differences in *Wolbachia* titer or gene expression. However, the lack of segmentation is known in natural contexts. Interestingly, the marked number of degraded cephalic furrow *wmk* embryos is proportional to the number of missing males in adult sex ratios (Fig 2). These results imply that the degraded embryos 16–17 h AED and the reduced sex ratios of surviving adults are the result of *wmk*-induced defects in early male embryos. Taken together, there are four key results: (i) *wmk* induces DNA defects 2–4 h AED, (ii) embryos arrest after cephalic furrow formation, (iii) embryos become degraded by late stages of embryogenesis, and (iv) embryonic defects lead to downstream reductions in sex ratios of surviving flies. Notably, the 2–4 h time window is when defects begin to significantly occur in *D*. *bifasciata*. The corresponding adult sex ratios for this experiment are shown in S5A Fig.

**Fig 4:**
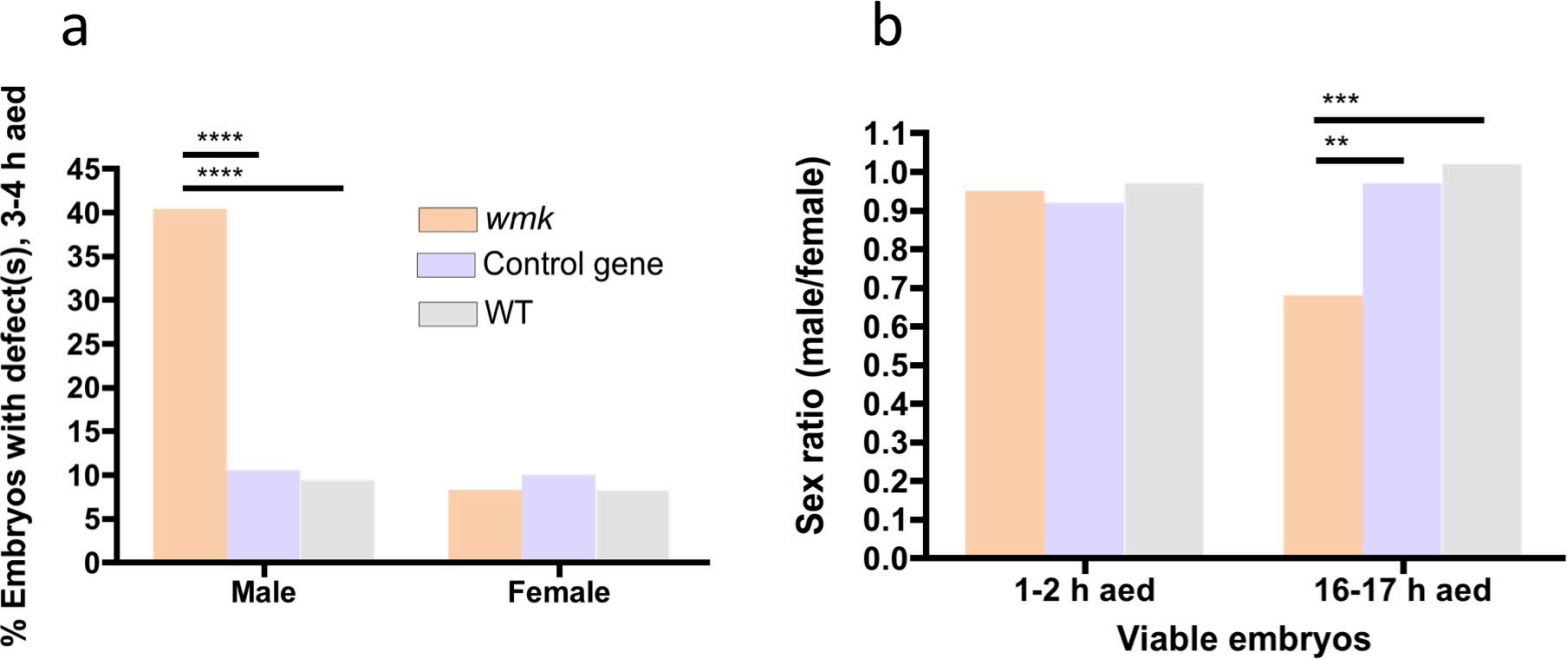
*wmk*-induced embryonic defects are enriched in males. Data are from pooled embryos (both sexes, expressing and non-expressing, see methods) with either *wmk*, the control gene, or a WT background. (A) Graph quantitating the percentage of 3–4 h AED embryos (males or females) that have at least one defect (*wmk* males N = 228, control gene males N = 190, WT males N = 170, *wmk* females N = 240, control gene females N = 200, WT females N = 158). (B) Graph quantitating the sex ratio of viable embryos (not degraded, no visible defects) across two development times (1–2 h *wmk*, N = 105 m, 111 f; 1–2 h control gene, N = 30 m, 141 f; 1–2 h WT, N = 112 m, 115 f; 16–17 h *wmk*, N = 104 m, 154 f; 16–17 h control gene, N = 116 m, 120 f; 16017 h WT, N = 110 m, 108 f). m = male, f = female. Statistics were performed with a Chi-square test comparing the three genotypes within each category (male or female in (A) and 1–2 h or 16–17 h in (B)). These experiments were performed once. ** p<0.01, *** p<0.001, ****p<0.0001.

Next, we confirmed that the cytological defects in embryos 3–4 h AED are male-biased using fluorescent *in situ* hybridization (FISH) with a DNA probe specific to the Y chromosome (S6 Fig, expressing and non-expressing embryos, see methods). 40% of male *wmk* embryos exhibit defects versus 9% of female *wmk* embryos and 9–10% of WT and control gene embryos (Fig 4A). In addition, while the embryonic sex ratios are not biased at 1–2 h AED, they are biased among viable (non-degraded) embryos fixed 16–17 h AED (Fig 4B), as expected. The corresponding adult sex ratio of 0.68 was similar to the embryonic sex ratio (S5B Fig), further indicating that male killing occurs during embryogenesis. These results specify that defects and degradation are enriched in males.

**Fig 5:**
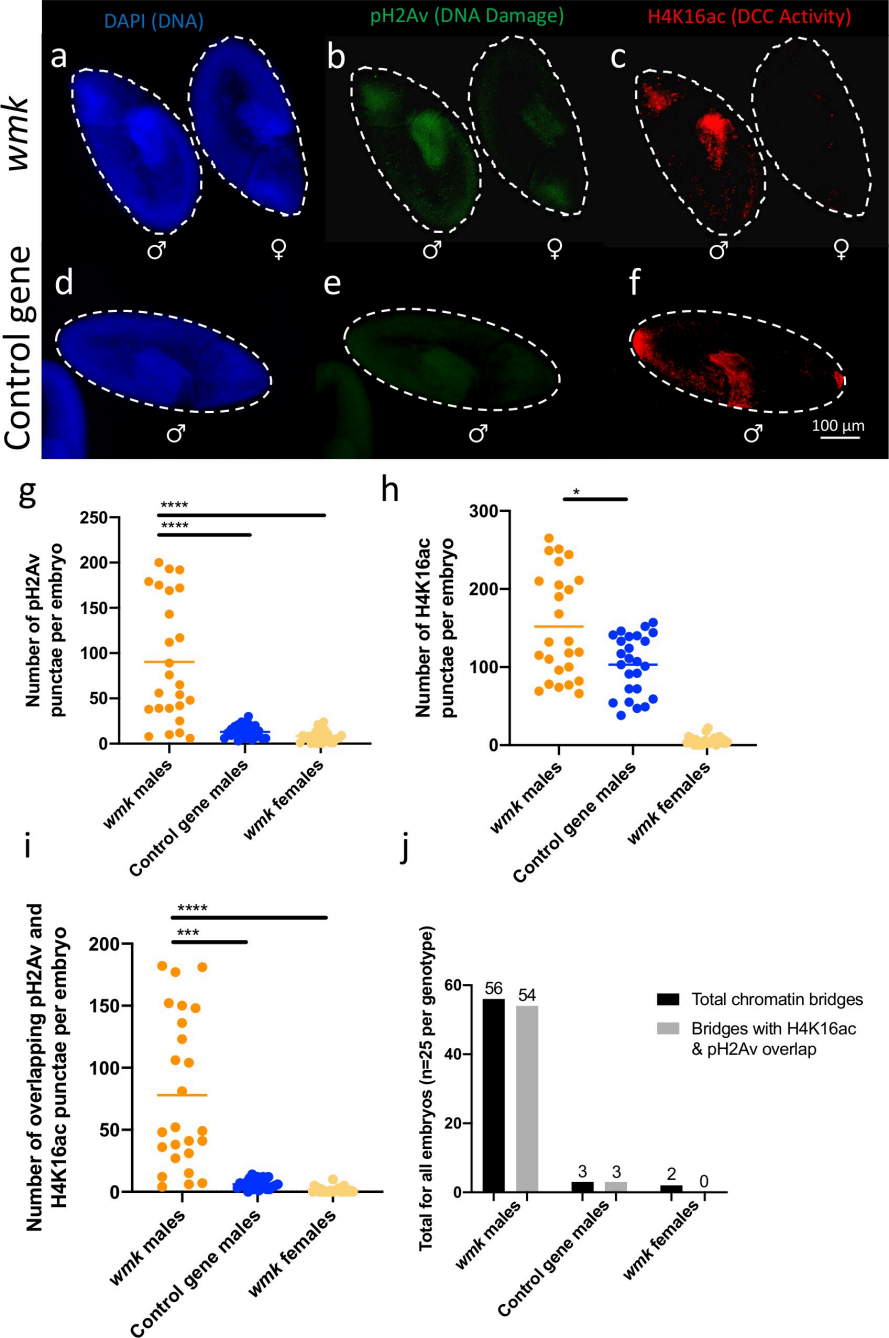
Transgenic expression of *wmk* causes DNA damage in association with H4K16ac. Images and data are from embryos 4–5 h AED expressing a transgene under the *arm* driver. (A) DAPI DNA stain of male and female embryos, side-by-side, expressing *wmk*. Sexes determined by H4K16ac antibody. (B) pH2Av antibody staining of the same embryos as (A). The male has distinct punctae or foci, while the female does not. All embryos exhibit either a low level of autofluorescence at the same wavelength as the secondary antibody (Alexa 488) visible in both embryos or there is background staining. (C) H4K16ac antibody staining of the same embryos as (A). Distinct punctae are only visible in males, while females can exhibit low levels of staining. (D) DAPI DNA stain of control gene male. Sex determined by H4K16ac antibody. (E) pH2Av antibody staining of the same embryo as (D), with no distinct punctae and only autofluorescence or background staining visible. (F) H4K16ac antibody staining of the same embryos as (D). (G) Graph of the number of pH2Av punctae visible in each embryo. N = 25 embryos per genotype. Statistics are based on a Kruskal-Wallis one-way ANOVA followed by Dunn’s correction. (H) Graph of the number of H4K16ac punctae visible in each of the same embryos as measured in (G). Statistics are based on a Mann-Whitney U test comparing the two male categories. (I) Number of cases where pH2Av punctae directly overlapped with H4K16ac punctae in the same embryos as (G) and (H). Statistics are based on a Kruskal-Wallis one-way ANOVA followed by Dunn’s correction. (J) Graph of the total number of chromatin bridges and the total number of bridges with overlapping H4K16ac and pH2Av punctae in each of the three genotypes measured in (G-I). All images were taken at 20X zoom. This experiment has been performed once. *p<0.05, ***p<0.001, ****p<0.0001.

To further determine the similarity in lethality between the transgenic *wmk* and natural infection phenotypes, we assessed embryos for an association between DNA damage and dosage compensation. In previous work, male *D*. *bifasciata* embryos infected with *Wolbachia* exhibited an accumulation of DNA damage in association with dosage compensation [49]. We assessed *wmk*-expressing and control embryos 4–5 h AED for the same association (Fig 5). Using the *armadillo* driver, we stained embryos with antibodies for pH2Av (phosphorylated histone H2Av, indicative of DNA damage) and H4K16ac (acetylation of histone H4 at lysine 16, primarily mediated on the X-chromosome by the male-specific dosage compensation complex or DCC). Males that express *wmk* have a greater number of pH2Av and H4K16ac punctae or foci than both *wmk*-expressing females and control gene-expressing males (Fig 5A–5H). The higher number of H4K16ac punctae may potentially reflect increased DCC activity in *wmk*-expressing embryos. An example set of images for a control gene female is shown in S7 Fig. In addition, a significantly higher proportion of the two types of punctae overlapped (Fig 5I). This suggests a mechanism of death related to DNA damage that is associated with dosage compensation, as with natural infections. Within males, there is a cohort of *wmk* embryos that have a higher number of H4K16ac and pH2Av punctae (Fig 5G and 5H). Interestingly, this proportion (~40%) is similar to the proportion of males that die according to adult sex ratios (S3A Fig). In addition, the H4K16ac and pH2Av punctae often overlapped with chromatin bridging, which is another phenotype previously observed in *D*. *bifasciata* [49]. The overlap happened more frequently in *wmk*-expressing males than females or control gene-expressing males (Fig 5J). Taken together, results demonstrate that DNA damage is accumulating at sites of dosage compensation activity in *wmk*-expressing embryos.

### *wmk* is expressed in *Drosophila* embryos infected with *Wolbachia*

**Fig 6:**
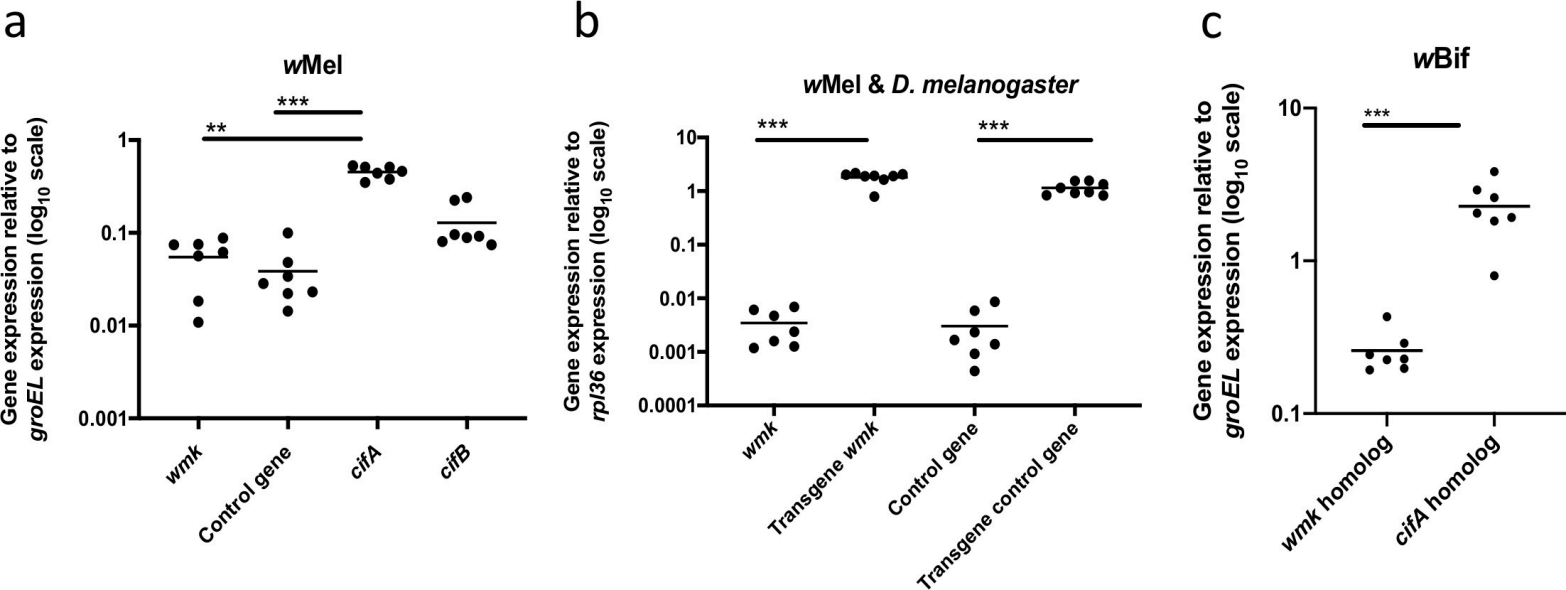
Native *Wolbachia* gene and transgene expression in embryos of *D*. *melanogaster* and *D*. *bifasciata*. (A) Graph of native prophage WO and *Wolbachia* gene expression in *w*Mel-infected *D*. *melanogaster* embryos fixed 4–5 h AED (pooled male & female) compared to *Wolbachia gro*EL. Each point (n = 7) represents a pool of 30 embryos from a set of 10 mothers and 2 fathers. (B) Graph of (i) transgene expression in uninfected *D*. *melanogaster* embryos fixed 4–5 h AED versus (ii) native gene expression in samples from a, both compared to *Drosophila rpl*36 (pooled male, female, expressing, and non-expressing for transgenes). Each point (transgene n = 8, native n = 7) represents a pool of 30 embryos from a set of 10 mothers and 2 fathers. (C) Graph of *w*Bif *Wolbachia* gene expression in *D*. *bifasciata* embryos 4–5 h AED (pooled male & female) compared to *Wolbachia groEL*. Homologs to the control gene in this study and *cifB* were not measured as they are not present in the *w*Bif genome assembly. Each point (n = 7) represents a pool of 30 embryos from a set of 10 mothers and 2 fathers. Values denote 2^-ΔCt^. Statistics are based on a Kruskal-Wallis one-way ANOVA followed by Dunn’s correction. This experiment has been done once. **p<0.01, ***p<0.001.

To establish a native expression profile for *wmk*, we measured relative transcription in *Wolbachia*-infected embryos fixed 4–5 h AED, which is the estimated time of death of most *wmk*-expressing male embryos. In *w*Mel-infected embryos, native *wmk* and control gene transcripts were approximately 10-fold lower than the highly expressed CI gene, *cif*A (Fig 6A). There were no significant differences with either gene compared to the less abundant *cif*B gene transcript. Also, expression levels of the *wmk* and control transgenes are similar in uninfected *D*. *melanogaster*, and both are expressed significantly higher than native bacterial transcription of the same genes (Fig 6B). Finally, *D*. *bifasciata* embryos infected with *w*Bif male-killing *Wolbachia* showed a *wmk*-like expression profile similar to *w*Mel, whereby the *cifA* homolog is expressed significantly higher than the *wmk* homolog (Fig 6C). This suggests that differences in *cifA* vs *wmk* gene expression do not account for differences in reproductive parasitism phenotype where both CI and male killing can be induced by the same bacterial strain. Phenotypic differences may instead be determined by another factor such as host genotype.

### The Wmk protein is a putative DNA-binding protein

Phyre2 protein modeling [50] predicts that Wmk from *w*Mel is globular and composed of α-helical secondary structures matching several transcriptional regulators, suppressors, and DNA-binding proteins (S8A Fig). The best match to known protein structures, based on both alignment confidence and sequence identity, is the *Salmonella* temperate phage Rep-Ant complex, a dimerized DNA- and peptide-binding repressor [51] (99.8% homology confidence, 19% sequence identity, S8B Fig). Wmk may function similarly as a bipartite protein where the dimers are physically connected, especially considering that single HTH domains typically dimerize and act as transcriptional regulators across domains of life [52]. Further, predicted structures of the Wmk homologs in *w*Bif (S8C Fig), *w*Inn/*w*Bor (same sequence, S8D Fig), and *w*Rec (S8E Fig) are all very similar to the structure from *w*Mel. Indeed, all exhibit a 5 α-helix bundle, connected by a long, flexible linker to another 4 α-helix bundle. This is despite wide variation in amino acid sequence (e.g., *w*Bif Wmk has a 26.2% amino acid sequence identity to *w*Mel Wmk, which represent the most distantly related protein pair). S6 Table shows amino acid pairwise percent identity between *w*Mel Wmk and homologs from known male-killers. This similarity in overall protein structure, despite sequence divergence, suggests that the homologs may retain the same general function with target(s) that are possibly divergent across host species, such as different DNA sequences of homologous genes. Wmk may also have another function that accounts for structural conservation despite sequence differences across divergent hosts.

To assess conservation in different regions of the protein, we also analyzed Wmk amino acid divergence across homologs, including that of *w*Bif and all homologs in S1 Fig. There is relatively high sequence conservation overall across the protein (S9A Fig), but there are two areas of high variability adjacent to the two HTH DNA-binding domains that may be important for functional differences across strains or hosts (S9B Fig). In addition, although there is lower variation across DNA-binding regions relative to other parts of the protein, there is still variability that could account for differing abilities of homologs to cause a phenotype in one host versus another.

## Discussion

This study reports twelve key results supporting *wmk* as a male-killing gene candidate: (i) *wmk* recurrently associates with genomic screens for reproductive parasitism; it is on the shortlists of candidate phage WO genes in *Wolbachia* male-killers and CI-inducers [29]. (ii) The *wmk* gene is found in all sequenced male-killers including the reduced phage WO genome of *w*Rec (which retains ~25% of the full phage WO genome) and the divergent phage WO genome of *w*Bif. (iii) *wmk* is common, divergent in sequence, and located in the eukaryotic association module of phage WO that is enriched with sequences predicted or known to contain eukaryotic function and homology [32]. In this region, *wmk* is a few genes away from the two causative cytoplasmic incompatibility genes, *cifA* and *cifB*, that modify arthropod gametes [29]. (iv) Transgenic expression of *wmk* consistently induces a sex-ratio bias, but the phenotype does not recapitulate other forms of reproductive parasitism. (v) No sex ratio bias results from expression of other transgenes tested thus far under the same expression system, making the phenotype specific to *wmk*. (vi) Canonical DNA defects are recapitulated under transgenic expression at the same time in development as natural systems. (vii) *wmk* is naturally expressed in *w*Mel and *w*Bif embryos at the time the defects are known to occur in *D*. *bifasciata*. (viii) The Wmk protein is predicted to interact with DNA when DNA defects are a hallmark of *Wolbachia* male killing. (ix) *wmk* is unique to *Wolbachia*, and the *Wolbachia* male-killing mechanism has some unique phenotypic features compared to other male-killers. For example, the dosage compensation complex is not mislocalized in *Wolbachia* infection, but it is in *Spiroplasma* infection [13, 49]. (x) The phenotype can be induced with drivers that yield approximately ten-fold variation in expression levels, indicating the highest *Act5c* levels of expression are not necessary for the phenotype. (xi) DNA damage is more common in *wmk* males than in controls and it is associated with H4K16ac, which parallels data in natural infections. (xii) Wmk’s predicted structure is conserved across arthropod hosts despite sequence divergence, indicating it likely has conserved function.

Investigations into putative microbial male-killing genes have largely been hampered by an inability to culture or genetically manipulate intracellular bacteria and their mobile genetic elements. Recently, the gene Spaid in the endosymbiont *Spiroplasma poulsonii* was identified as a likely candidate underpinning killing of *D*. *melanogaster* males, possibly through misregulation of male dosage compensation [28]. Indeed, dosage compensation is an identified host target in *Spiroplasma* male killing [53, 54], and may be involved in *Wolbachia* male killing as well, although likely through a different method such as increased activity rather than mislocalization that is typical of *Spiroplasma* infection [49]. It also appears that the *wmk*-mediated mechanism of male death may involve dosage compensation, as it recapitulates H4K16ac associations with DNA damage, but this remains to be confirmed with further experiments. Interestingly, *wmk* males have slightly more H4K16ac than their control gene counterparts, raising the possibility that death is correlated with either accelerated or a greater amount of H4K16ac. Whether this is true and whether the dosage compensation complex is directly or indirectly involved both remain to be determined.

Spaid is on a plasmid and has no homologs in *Wolbachia*, though it was previously noted that locus WD0633 in *w*Mel has similar protein domains consisting of ankyrin and OTU domains [28]. However, WD0633 was not predicted here to be on the shortlist of candidates for *Wolbachia* male-killing due to its absence in *w*Rec. *wmk* is also in the genome of a mobile element (phage WO), likely originated in *Wolbachia*, and has no homologs in *Spiroplasma*. This indicates that there could be an emerging trend of endosymbiotic reproductive parasitism genes and candidates in mobile elements (including the *cifA* and *cifB* phage WO genes for CI). Both Spaid and *wmk* exhibit independent origins from each other. This finding is consistent with arguments that differences in observed male-killing phenotypes and sex determination systems of affected hosts may be due to distinct male-killing genes and/or mechanisms [55]. Other male-killing candidate genes may also exist. If so, they could support the observation that male killing can independently arise in bacterial symbionts. Identification of additional genes and comparisons of their mechanisms is an important area of future work.

Wmk is also a putative DNA-binding transcriptional regulator (S8 Fig), which is notable in light of previous studies demonstrating *Wolbachia*’s ability to modulate host transcription to induce various phenotypes. For example, *Spiroplasma* [53, 54] and likely *Wolbachia* [49], kill males through the host dosage compensation complex, which is a critical mediator of transcriptional differences between male and female sex chromosomes. These reproductive parasites are therefore likely interfering with regulatory processes for host gene expression in males, which is a likely cause of male death. In addition, *Wolbachia* influences on host transcription have been implicated in the CI phenotype [56] and virus inhibition [57, 58]. As *wmk* transgene expression similarly leads to DNA damage correlated with dosage compensation, it may follow a trend in the field of *Wolbachia* affecting the regulation or deregulation of host gene expression.

If *wmk* is the causative agent of male killing, then the *w*Mel genome could be multipotent and able to induce different phenotypes (e.g., CI and male killing) either in other hosts or under different environmental conditions. This premise remains to be evaluated in future studies. Assuming *wmk* is a bona fide male-killing gene, then some patterns about multipotency emerge. First and as noted earlier, *w*Mel and *w*Rec from *D*. *recens* are very closely related *Wolbachia* strains and have a 99.7% genome-wide identity [35]. Importantly, *w*Rec is a known multipotent strain that causes CI in its native host and male killing in a sister species [22]. While its genome has lost many prophage WO genes, it retains *wmk* and the *cif* genes that may underpin its multipotency, similar to *w*Mel. Second, while CI genes and phenotype often correlate, *wmk* is not always associated with male killing. *wmk* and its homologs are present in all sequenced male-killers, and they are also common in many other strains not known to cause male killing (Fig 1, S1A Fig). In *w*Mel and potentially other strains, lack of male killing in native hosts is possibly due to host resistance to male killing, as is likely in *D*. *recens* [22]. Importantly, host suppression of male killing is common [16, 21, 22, 37, 38], presumably because of the evolutionary pressure on the host to develop a counter-adaptation that avoids extinction. Therefore, though the *wmk* gene is more common than the male-killing phenotype, this would be expected if the frequency of resistance is indeed high. It is also possible that male killing is a multilocus trait that requires another gene to induce the phenotype in its natural context. Moreover, differences in *Wolbachia* titers, and/or insufficient expression of native *wmk* within *D*. *melanogaster* may contribute to the lack of male killing by *w*Mel, however this is unlikely given the similarly lowly-expressed *wmk* homolog in the *w*Bif male-killing strain. Finally, *wmk* and the *cif* genes are similarly disrupted, degraded, or lost in parthenogenesis-inducing *Wolbachia* strains *w*Uni from *Muscidifurax uniraptor* wasps, *w*Tpre from *Trichogramma pretiosum* wasps, and *w*Fol from *Folsomia candida* springtails. Therefore, multipotency is interestingly common for CI and male killing and will resultantly be rare in parthenogenesis strains.

There is considerable amino acid sequence divergence in Wmk homologs across several arthropod orders that harbor male-killing *Wolbachia*. One potential reason for the divergence is that if a single gene kills many or all of these hosts in nature, a premise which remains to be evaluated, it may be divergent due to selection to target the varied genetic and cellular bases of sex determination in these hosts. Second, if there is a single gene behind the phenotype, it could explain the relatively high frequency of host resistance since hosts would counter-adapt to one gene product rather than multiple products. Under antagonistic coevolution, *wmk* would evolve to kill males, the host adapts to resist the male killing, and *wmk* would follow suit and adapt again, continuing the evolutionary arms race. Third and in addition to coevolutionary bouts of *wmk* adaptation and host counter-adaptation, pleiotropy or multiple functions of *wmk* could also explain the sequence divergence in *wmk* homologs, especially in hosts that do not exhibit male killing.

Identification and further investigation of male-killing genes have relevance to translational applications in pest or vector control as male killing can theoretically be used in population suppression to crash target populations. Population modeling indicates that use of male killing in conjunction with other population-crashing techniques such as the Sterile Insect Technique (SIT), where sterilized males are released to compete with fertile males, could decrease the time to crash the population and increase the chances of success [26]. In this context, male killing genes might be used to transform an endosymbiotic microbe or host to either add or enhance male-killing ability. Alternatively, a male-killing infection could be established in a host where one does not natively exist. These techniques may be desirable in cases of invasive species of disease-carrying mosquitoes or agricultural pests. Techniques like SIT can fail if males are not completely sterile or because of reduced mating competitiveness with fertile males [59, 60]. Therefore, a two-pronged approach to simultaneously reduce viable matings in the wild (SIT) while killing off males (male killing) could in principle be used to more effectively crash populations prone to SIT failure on their own [26], although this remains to be empirically evaluated.

There are many remaining questions for the future, including ones that are important for understanding a male-killing gene’s role in host evolution and its potential in pest or vector control. First, is the *wmk* candidate gene in *Wolbachia* required for the phenotype in natural contexts? In the absence of the ability to knock out genes, it cannot yet be absolutely stated if *wmk* is used by bacteria to kill males in nature. Therefore, in addition to the transgenic expression, phenotype recapitulation, and sequence analyses demonstrated thus far, knocking out these genes in their resident genome will be important to assessing a change in phenotype. Second, can *wmk* homologs from related symbiont strains kill males? This will involve testing homologs in a genetically tractable host. Third, what is the exact mechanism of Wmk-induced male death? As *wmk* is annotated as a transcriptional regulator, it may act by controlling host transcription in a way that harms males. In addition, results indicate that the mechanism may involve dosage compensation. Fourth, what is the reason that transgenic *wmk* expression does not kill all males? Is it host resistance, inadequate expression patterns, divergence in host target or bacterial toxin gene sequence, or is another gene involved? We have tested a likely gene partner (WD0625) and multiple expression drivers (*Act5c*, *nanos*, *arm*, and MTD) to assess this, however no attempts so far have yielded answers. Finally, applications of male-killing bacteria or the genes to vector and pest control remain to be explored beyond population genetic theory [26].

The discovery of *wmk*-induced male death advances an understanding of the genes in the eukaryotic association module of prophage WO that interact with animal reproduction [29]. Moreover, male-specific lethality naturally occurs in many arthropods and has important influences on arthropod evolution [16, 19, 22, 23, 61, 62], such as modifying mate choice and selecting for male resistance to the phenotype [11, 55]. Male killing may also serve as a means to enhance population suppression methods for vectors or pests [26]. Thus, assessing male-killing gene candidates advances an understanding of the tritrophic crosstalk between phages, reproductive parasitic bacteria, and animals as well as their potential in arthropod control programs [24, 26].

## Materials and methods

### Experimental design

Most *Drosophila* experiments (unless otherwise noted) were set up with the following design. Crosses in each experiment were conducted by mating 10 female heterozygous *Act5c*-Gal4/CyO driver flies to 2 male homozygous transgene flies (both uninfected, unless otherwise noted; switching the gender for each genotype does not alter the effect). The offspring of these crosses were used for all experiments, except where noted. As the *Act5c*-Gal4/CyO driver strain is heterozygous, when driver flies are crossed to homozygous transgene flies, half of the offspring express the gene (those that inherit the *Act5c* driver gene that produces the Gal4 transcription factor), while the other half do not (those that inherit the CyO chromosome, which does not produce Gal4). Therefore, expressing males, expressing females, non-expressing males, and non-expressing females are expected in equal proportions under Mendelian inheritance. These four genotypes can only be visibly assessed in adulthood. Visually, embryos cannot be distinguished (except when fixed for microscopy with the Y chromosome FISH probe, when sex can be distinguished), while larvae can only be differentiated by sex.

Alongside several experiments, including the cytology in Figs 3 and 4, sex ratios were measured concurrently. When flies were set up in the crosses described above, siblings were also set up in vials with CMY media. The protocol to measure sex ratios was then followed to obtain sex ratios side by side with these experiments. The results are in the extended data, where noted.

The maternal triple driver (MTD) was tested by crossing this homozygous driver strain to homozygous transgene flies in the same design as above. This crossing leads to transgene expression in all offspring because the driver is homozygous. Females expressing the transgene in their ovaries (MTD leads to targeted gene expression in the germline, specifically by loading embryos with the product) were then crossed to WT flies. Offspring were then quantified to measure sex ratios.

### Comparative genomics and evolutionary analysis

Putative Wmk domains were identified by a CD-SEARCH of NCBI’s Conserved Domain Database (https://www.ncbi.nlm.nih.gov/Structure/cdd/wrpsb.cgi). For the full-length analysis (S1A Fig), homologs were identified by a BLASTn of NCBI’s nucleotide collection (nr/nt) and whole genome shotgun sequence (wgs) databases. The sequences reported were reciprocal best BLAST hits with *w*Mel *wmk*. Partial sequences and/or those located at the end of a contig were excluded from downstream analysis. For the comparative genomic analysis, *wmk*, *cif*A, and *cif*B homologs were identified by manual annotations of prophage WO regions within known male-killing strains. Homology was confirmed by translating each gene and performing a BLASTP search against *w*Mel in NCBI. Only sequenced male-killing *Wolbachia* genomes in *Drosophila* were compared to demonstrate homologs clustering with gene synteny (S1B Fig). For both phylogenetic analyses, sequences were aligned using the MUSCLE plugin in Geneious Pro v8.1.7 and all indels were stripped. Trees were built using the MrBayes plugin in Geneious and were based on the best models of evolution, according to the corrected Akaike Information Criteria (AICc), as estimated by JModelTest and ProtTest v3.4.2, respectively. The models each predicted the GTR+I+G model for S1A Fig and the JTT+G model for S1B Fig, respectively. *w*Bif was excluded due to high sequence divergence. Protein modeling was performed with Phyre2 [50].

For the male-killer comparative genomics analysis, the entire *w*Bif draft assembly was searched for prophage WO-like regions. Five WO-like islands were found, and the genes in these regions were annotated using the NCBI BLASTP and conserved domain database. We then performed a 1:1 BLASTP of the annotated genes against query genomes. If it was present in *w*Bif, the *w*Rec, *w*Inn, and *w*Bor genomes were searched for homologs, in the given order. If the gene was absent in one strain, it was marked as absent and excluded from further analysis. Genes were removed if they were: (i) absent in one or more of the strains (*w*Bif, *w*Rec, *w*Inn, and *w*Bor), (ii) mobile elements (including IS elements, reverse transcriptases of group II intron origin, or recombinases), (iii) disrupted genes (frameshift with early stop codons) in one or more of the strains, and, (iv) if the E-value was less than E-20. See S1 Table for a list of all removed genes along with rationale for exclusion.

### *Wolbachia* gene sequencing

The *D*. *innubila Wolbachia* genome was sequenced from a single wild-caught female. Briefly, *D*. *innubila* were captured at the Southwest Research Station in Arizona over baits consisting of store-bought white button mushrooms (*Agaricus bisporus*). DNA was extracted using the Qiagen Gentra Puregene Tissue kit (#158689, Germantown, Maryland, USA). A genomic DNA library was constructed for several individuals using a modified version of the Nextera DNA Library Prep kit (#FC-121-1031, Illumina, Inc., San Diego, CA, USA) reagents [63]. DNA from an infected female was sequenced on a fraction of an Illumina HiSeq 2500 System Rapid-Run to generate 14873460 paired-end 150 base-pair reads. Reads were aligned to a draft *D*. *innubila* genome and all non-aligned reads were assembled *de novo* using Spades [64]. Those contigs blasting to other *Wolbachia* accessions were retained as putative *Wolbachia* genomic contigs.

The *Wolbachia* genomes of *w*Bif and *w*Bor were sequenced from *D*. *bifasciata* (line bif-F-MK [65]) and *D*. *borealis* (line PG05.16 [39]) respectively. Following the protocol developed in Ellegaard et al. [66], *Wolbachia* cells were purified from ~20 freshly laid (less than 2 hours) and bleach-dechorionated embryos by homogenizing them in phosphate-buffered saline solution (PBS) and conducting a series of centrifugation/filtration steps as explained in Ellegaard et al [66]. A multiple-displacement amplification was carried out directly on the bacterial pellet using the Repli-g midi kit (Qiagen). The amplified DNA was cleaned with QIAamp DNA mini kit (Qiagen). From each sample, both 3kb mate-pair and 50 bp paired-end DNA libraries were prepared and sequenced on a 454 Roche FLX (Department of Biochemistry, Cambridge, UK) and Illumina HiSeq2000 instruments (The Genome Analysis Center, Norwich, UK) respectively. The sequencing generated 203,565 and 239,485 454 mate-pair reads as well as 35,415,012 and 30,624,138 Illumina reads for *w*Bif and *w*Bor respectively. De novo hybrid assemblies combining 454 reads and a 10% subset of the Illumina reads were performed in Newbler (454 Life Sciences Corp., Roche, Branford, CT 06405, US). Contigs blasting to other *Wolbachia* accessions were retained as putative *Wolbachia* genomic contigs. Scaffolds were extended to fill regions with “N“s using GapFiller v.1-11 [67].

The *Wolbachia* genome of *D*. *innubila* (*w*Inn) was sequenced by the R. Unckless lab. The *Wolbachia* genomes of *D*. *bifasciata* (*w*Bif) and *D*. *borealis* (*w*Bor) were sequenced by the F. Jiggins lab. The genomes will be published by the respective contributors at a later date, and only the phage WO gene regions involved in this publication are publicly available (the regions in Fig 1).

### *Drosophila* strains

The *Wolbachia* transgene strains were generated as described previously [29]. WD0626 (*wmk*) and WD0034 (control gene) were both inserted into an attP site in the BSC8622 (WT) line of genotype *y*^1^*w*^67c23^; P[CaryP]P2 obtained from the Bloomington Drosophila Stock Center. WD0625 was inserted into the BSC9723 strain, with a genotype of *y*^1^M[vas-int.Dm]ZH-2A w*; PBac[y+-attP-3B]VK00002. WD0508 was inserted into the *y*^1^M[vas-int.Dm]ZH-2A w*; P[CaryP]attP40 line. The genes were inserted into various strains to facilitate creation of strains that contain more than one gene homozygously. The *Act5c*-Gal4/CyO driver line is the same background as BSC3953, which is *y*^1^w*; P[Act5C-GAL4-w]E1/CyO. The maternal triple driver (MTD) strain BSC31777, genotype P[w[+mC] = otu-GAL4::VP16.R]1, *w*[*];P[w[+mC] = GAL4-nos.NGT]40; P[w[+mC] = GAL4::VP16-nos.UTR]CG6325[MVD1], was provided by J. Nordman. The *nanos*-Gal4 strain used in S4B and S4C Fig was previously described [29]. The *arm*-Gal4 driver strain BSC1560 is w[*]; p[w[+mW.hs] = GAL4-arm.S]11. The infected *D*. *bifasciata* flies were provided by G. Hurst and are infected with male-killing *Wolbachia*. The male-killing flies are maintained with males from a concurrently reared uninfected line also provided by G. Hurst.

### *Drosophila* rearing

*D*. *melanogaster* were reared on 4% cornmeal (w/v), 9% molasses (w/v), 1.6% yeast (w/v) (CMY) media. The flies developed at 25°C at 80% humidity with a 12 h light/dark cycle. Virgin flies were stored at room temperature after collections. During virgin collections, stocks were maintained at 25°C during the day and at 18°C at night. *Wolbachia*-uninfected transgene or driver lines were generated via tetracycline treatment of infected lines as described previously [29]. *D*. *bifasciata* are maintained on CMY media at room temperature.

### Sex ratio measurements

To assess the ability of the gene candidates to alter sex ratios, twenty replicates of 10 uninfected, 4–7 day old female driver flies and 2 uninfected, 1–2 day old male transgene flies were set up in vials with CMY media. They were left on the media to lay eggs for 36 h at 25°C, at which point adults were discarded. Once the offspring emerged, they were scored for both sex and expression or non-expression (if applicable), which was determined by presence or absence of the CyO wing phenotype as well as with eye color markers associated with *Act5c*-Gal4 and the transgene insertion. Any vials with fewer than 50 adult offspring were removed from the analysis, as this indicates either poor egg laying or abnormally low egg hatching (average = 120 offspring).

### Hatch rate

Extended data hatch rates (S4B and S4C Fig) were performed as previously described with the *nanos*-Gal4 driver [29]. The *nanos* driver was used to test induction of CI instead of *Act5c*-Gal4/CyO because it is expressed more specifically in the gonads where CI is induced [29].

### Embryo cytology

For Figs 3 and 4, eight stock bottles were set up per genotype, each with 60 uninfected, 4–7 day old *Act5c*-Gal4/CyO females and 12 uninfected, 1–2 day old transgene or WT males. Grape juice agar plates, made as described previously [29], with a small amount of baker’s yeast (Red Star) placed on each bottle opening and fixed with tape. They were then placed with the grape plate down in a 25°C incubator overnight (~16 hr). The grape plates were then replaced with fresh plates and fresh yeast. The flies were then allowed to lay eggs in 1 h increments, replacing the previous plates with fresh ones each time. They were then allowed to sit at room temperature for 1 h (embryos 1–2 h old), 3 h (3–4 h old), or 16 h (16–17 h old). Once they had reached the desired point in development, the embryos were fixed and stained, using a slight modification of the protocol outlined by Cheng et al. 2016 [13]. Briefly, the embryos were dechorionated in 50% bleach and fixed for 15 minutes in a 1:1 4% paraformaldehyde:heptane mixture while shaking on a tabletop vortexer at about 150 rpm. The solution was discarded, and the embryos were then devitellinized in a 1:1 heptane:methanol mixture by shaking vigorously for one minute. The solution was removed, and the embryos were placed in fresh methanol and stored at 4°C until the next steps were done, at least 16 h later. Then, the methanol was removed and the embryos were rehydrated in a series of methanol:water solutions, in the order of 9:1, then 1:1, then 1:9, each for 15 minutes while mixing on a Nutator. They were then treated with 10 mg/mL RNase A (Clontech Labs) by incubating them at 37°C for 2–3 hr with enough RNase solution to cover the embryos. Once the RNase was removed, the embryos were washed three times for 5 min each in PBST (1X PBS, 0.1% Tween 20), while mixing on the Nutator. They were then re-fixed in 4% paraformaldehyde for 45 minutes with mixing and were then washed or incubated with several solutions with mixing on the nutator. First, they were washed three times in saline-sodium citrate/Tween 20 buffer (SSCT, 2X SSC buffer, 0.1% Tween 20) for 10 minutes each. They were then incubated with a series of SSCT/formamide solutions for 10 minutes each in the following order: 80% SSCT/ 20% formamide, 60% SSCT/ 40% formamide, 50% SSCT/ 50% formamide. Then fresh 50% SSCT/ 50% formamide was added and the embryos were incubated at 37°C for 1 h. The solution was removed, and the embryos were then hybridized with the Y-chromosome FISH probe. This was done by mixing 36 μL FISH hybridization solution (1g dextran sulfate, 1.5 mL 20X SSC, 5 mL formamide, to 15 mL with DNase-free water) [68], 3 μL DNase-free water, and 1 μL 200 ng/μL Y-chromosome FISH probe (sequence 5’-AATACAATACAATACAATACAATACAATAC-3’ synthesized with Cy5 conjugated to the 5’end (IDT)) using the sequence published by Cheng et al. 2016 [13]. Hybridization was done in a thermocycler by denaturing at 92°C for 3 min, followed by hybridizing at 37°C overnight (~16 h). Then, the embryos were again washed in a series of solutions on the nutator. They were done in the order of three 15 min 50% SSCT / 50% formamide washes, one 10 min 60% SSCT / 40% formamide wash, one 10 min 80% SSCT / 20% formamide wash, and three 10 min SSCT washes. They were then mounted on glass slides with ProLong Diamond Antifade (Life Technologies, P36970) mounting media that contained 1 μg/mL propidium iodide (Sigma Aldrich).

Imaging was performed at the Vanderbilt University Cell Imaging Shared Resource (CISR) with a Zeiss LSM 510 META inverted confocal microscope. Images are of a single plane. Image analysis and preparation was done with ImageJ software. Image brightness and contrast were adjusted for visibility, but adjustments were applied equally across each whole image.

For Fig 5, a different fixing and staining protocol was used. Eight bottles were set up per genotype with 60 uninfected *armadillo(arm)*-Gal4 females crossed to 12 uninfected *wmk* or control gene males with a small amount of baker’s yeast (Red Star) placed on each bottle opening and fixed with tape. They were then placed with the grape plate down in a 25°C incubator overnight (~16 hr). The grape plates were then replaced with fresh plates and fresh yeast. The flies were then allowed to lay eggs in 1 h increments, replacing the previous plates with fresh ones each time. They were all aged to 4–5 h AED. Once they had aged to the desired point in development, they were fixed and stained using the protocol described in Hall & Ward [69]. Embryos were dechorionated for 2 min in 50% bleach and rinsed with water. They were then fixed with shaking in 1:1 4% paraformaldehyde to heptane at room temperature for 20 min. The bottom paraformaldehyde phase was removed and methanol was added in equal volume to the remaining heptane and embryos. They were then devitellinized by shaking vigorously for 20 s. Embryos were stored in methanol at 4°C until staining. Staining was performed by first removing the methanol and rinsing with 750 μL blocking solution (Vector Laboratories Animal-Free blocking solution SP5030). The embryos were then rinsed in 1X PBS twice. The PBS was removed and the embryos were permeabilized in 750 μL blocking solution for 30 min at room temperature with rocking. The blocking solution was removed and the embryos were rinsed with 1X PBS once. The embryos were then incubated with primary antibodies in 500 μL blocking solution overnight at 4°C with rocking. The antibodies included histone H2AvD pS137 antibody (1:100, Rockland 600-401-914), anti-acetyl-histone H4 (Lys16) antibody or H4K16ac (1:100, Millipore Sigma 07–329), and Sxl antibody (1:20, DSHB M18). The Sxl antibody developed by P. Schedl was obtained from the Developmental Studies Hybridoma Bank, created by the NICHD of the NIH and maintained at The University of Iowa, Department of Biology, Iowa City, IA 52242. In cases where primary antibodies were raised in the same animal, sequential staining was performed. After overnight staining with one antibody, the steps were repeated beginning with the initial blocking step for the second antibody.

After overnight staining, the embryos were washed in 1X PBS three times at room temperature with rocking for 5 min each. They were then incubated with 750 μL blocking solution for 30 min at room temperature with rocking. The blocking solution was removed and the embryos were rinsed in 1X PBS once. The embryos were then incubated with secondary antibodies in 500 μL blocking solution at room temperature with rocking for 1 h out of the light (all subsequent steps are also out of the light). The antibodies included goat anti-mouse IgG with Alexa Fluor 647 (1:500, abcam ab150115), goat anti-rabbit IgG with Alexa Fluor 594 (1:500, Invitrogen A11037), and goat anti-rabbit IgG with Alexa Fluor 488 (1:500, Invitrogen A11034). The embryos were then washed three times with 1X PBS at room temperature with rocking for 5 min each. They were then incubated with 750 μL blocking solution for 30 min at room temperature with rocking. The embryos were then rinsed once in 1X PBS. The embryos were then stained with 1μg/mL DAPI (Invitrogen D1306) for 10 min with rocking at room temperature. Embryos were then washed three times in 1X PBS for 10 min each with rocking at room temperature. They were then mounted on glass slides with ProLong Diamond Antifade (Life Technologies, P36970) mounting media.

Imaging was performed using a Keyence BZ-X710 Fluorescence Microscope and all images are a single plane. Images were taken at 20X magnification. Quantification of punctae was done by manually focusing on several planes that encompassed all punctae and quantifying punctae with overlapping signals. Images were analyzed using Keyence analysis software. Image brightness and contrast were adjusted and dehazing software was used for visibility, but adjustments were applied equally across each whole image.

### Gene expression

Gene expression in embryos from Fig 6 was measured in each of four groups. Group 1 was generated in crosses between *Act5c*-Gal4/CyO uninfected females crossed to *wmk* uninfected males. Group 2 was generated in crosses between *Act5c*-Gal4/CyO uninfected females crossed to control gene uninfected males. Group 3 was generated by crossing *y*^1^w* infected females to *y*^1^w* uninfected males. Group 4 was generated by crossing *w*Bif-infected *D*. *bifasciata* females to uninfected *D*. *bifasciata* males. Gene expression for S3B Fig was set up using two groups with either *Arm*-Gal4 or *Act5c*-Gal4/CyO uninfected females crossed to *wmk* males. For each group, 8 bottles were set up with 10 females and 2 males. A grape juice agar plate [29] with yeast was placed in each bottle. These were placed in a 25°C incubator overnight (16 h) for *D*. *melanogaster* or kept at room temperature (23°C) for *D*. *bifasciata*. Then, the plates were swapped with fresh ones. The flies were allowed to lay eggs for 1 h. The plates were then left at 25°C or 18°C for an additional 4 h to age them to be 4–5 h old (the estimated time of male death in *wmk* crosses). Embryos were then gathered in groups of 30 (each group from the same bottle) and flash frozen in liquid nitrogen. RNA was extracted using the Direct-zol RNA MiniPrep Kit (Zymo), DNase treated with DNA-free DNase (Ambion, Life Technologies), cDNA was generated with SuperScript VILO (Invitrogen), and RT-qPCR was run using iTaq Universal SYBR Green Mix (Bio-Rad). qPCR was performed on a Bio-Rad CFX-96 Real-Time System. Primers are listed in S4 Table. Conditions were as follows: 50°C 10 min, 95°C 5 min, 40x (95°C 10 s, 55°C 30 s), 95°C 30 s. For each gene measured, a standard curve was produced with known concentrations alongside samples with unknown concentrations. Primers are listed in S3 Table. Differences in gene expression were done by calculating 2^-Δct^ (difference in ct values of two genes of interest).

Confirmation of gene expression in adults from S2C and S2E Fig was done similarly. Samples were obtained by flash freezing adult offspring laid by siblings of the flies used in S2A Fig. Samples from S2B Fig were from pooled, whole-body extractions from three males of each genotype. Samples from S2C Fig were from pooled, whole-body extractions from three females of each genotype. Samples from S2E Fig were from pooled, dissected ovaries of six adult female siblings of flies of flies used in S2E Fig for each genotype. Samples were flash frozen in liquid nitrogen and then was processed (RNA extraction, DNase treatment, and cDNA treatment) as above. PCR was performed against positive controls (extracted DNA), negative controls (water), RNA, and cDNA. Gel image brightness and contrast were adjusted for visual clarity, but adjustments were applied equally across each whole image.

### Protein conservation

Protein conservation was calculated with the Protein Residue Conservation Prediction Tool [70]. Amino acid sequences from S1 Fig along with the *w*Bif Wmk homolog sequence were aligned using a MUSCLE alignment in Geneious Prime version 2019.1. This alignment was uploaded to the prediction tool with the following settings: Shannon entropy scores, a window size of zero, and no sequence weighting. Conservation values were then input into GraphPad Prism version 8 for visualization. HTH regions were indicated using the amino acids predicted to be in the domains according to the NCBI annotation of *w*Mel Wmk.

## Statistical analyses

Statistical analyses were done using GraphPad Prism software (version 5 or 8) or GraphPad online tools, unless otherwise noted. For comparisons among only two data categories, we used the two-tailed, non-parametric Mann-Whitney U test. For comparisons with more groups, a non-parametric Kruskal-Wallis one-way analysis of variance was used, followed by Dunn’s test for multiple comparisons, if significant. In cases of comparisons among groups where only a single measurement was taken per group (such as cytology experiments), a Chi-square test was used. Exact tests used and other important information are listed in the figure legends of each experiment.

## Supporting information

S1 Fig Comparative genomics of the *wmk* gene and protein. (A) Phylogeny of full-length *wmk* gene, based on an 893-bp alignment and GTR+I+G model of evolution. Full-length *wmk* homologs are widespread throughout prophage WO-containing *Wolbachia* strains, some of which are male-killing strains. Like many WO-associated genes, including CI factors *cifA* and *cifB*, the *wmk* phylogeny does not support evolution with the *Wolbachia* chromosome because homologs in A- and B-*Wolbachia* do not cluster according to supergroup. *Wolbachia* supergroups are illustrated as either black (A-*Wolbachia*) or red (B-*Wolbachia*) branches. *wmk* (WD0626) and homologs from male-killing strains are highlighted in cyan. Consensus support values are shown on the branches. The tree includes all taxa that are reciprocal best hits of *w*Mel *wmk*. See S5 Table for accession numbers and BLASTn E-values. (B) A Bayesian phylogeny of Wmk protein and homologous peptides from *w*Mel and sequenced male-killing strains in *Drosophila*, based on a 168 aa-alignment using the JTT+G model of evolution. It shows that homologs in these taxa cluster according to gene synteny within prophage WO genomes (see Fig 1). Consensus support values are shown on the branches. Colors correspond to Fig 1. Accession numbers and BLASTP E-values are provided in S4 Table.
(TIF)

S2 Fig *Act5c* and MTD expression of transgenes other than *wmk* do not cause a sex ratio bias. (A) Sex ratios were quantified for *wmk*, the control gene (WD0034), WD0625, WD0508, or dual *wmk*;WD0625 transgenes expressed with the *Act5c*/CyO driver. Expressing and non-expressing flies of each genotype are siblings. Each point represents the offspring of one vial of 10 mothers and 2 fathers. A biased sex ratio only results when *wmk* is expressed. Average N per vial is 78. Statistics are based on a Kruskal-Wallis one-way ANOVA followed by Dunn’s correction with only the non-expressing flies or only the expressing flies. Groups labeled “a” are significantly different compared to groups labeled “b”. Non-expressing flies are non-significant. Bars represent the average sex ratio. E = expressing, NE = non-expressing, *Act5c* = *Act5c* gene present, CyO = CyO chromosome present. This experiment has been performed once. (B) Transgenes are expressed in *Act5c* (E) adult males but not their CyO (NE) brothers as demonstrated by cDNA generated from males. Samples were taken from offspring of parental siblings from the experiment in (A). Samples were from pooled, whole-body, adult extractions of three individuals from each genotype. (C) Transgenes are expressed in *Act5c* (E) females, but not their CyO (NE) sisters as demonstrated by cDNA generated from females. See (B) for details. Both (B) and (C) have been performed once. (D) Sex ratios were similarly quantified for the listed transgenes using a maternal triple driver (MTD) where expression was driven in the mother throughout oogenesis and offspring were laid with the expressed products loaded into the eggs. Each point represents the offspring of a vial of 10 mothers and 2 fathers. Average N per vial is 74. Statistics are based on a Kruskal-Wallis one-way ANOVA followed by Dunn’s correction. Bars represent the average sex ratio. This experiment has been done once. (E) Transgenes are expressed in all adult offspring from the MTD driver as demonstrated by cDNA generated from siblings of mothers from the experiment in (D). Samples are from pooled, dissected ovaries of six flies. This experiment has been performed once. The meanings of notations in the gels are as follows: “dual *wmk*” indicates *wmk*;0625 co-expressing flies measured with *wmk* primers, “dual 0625” indicates *wmk*;0625 co-expressing flies measured with 0625 primers, “C” indicates cDNA, “R” indicates RNA, “-” indicates negative control, “+” indicates positive control.
(TIF)

S3 Fig The *wmk* phenotype can be induced with both the *arm* and *Act5c* drivers despite differences in expression levels. (A) Sex ratios were quantified for *wmk*, the control gene (WD0034), and WT flies. The *armadillo* (*arm*) driver is homozygous, so all offspring express the gene. Each point represents the offspring of one vial of 10 mothers and 2 fathers. A biased sex ratio results only when *wmk* is expressed, as with *Act5c* (Fig 2). Average N per vial is 73. Statistics are based on a Kruskal-Wallis one-way ANOVA followed by Dunn’s correction. This experiment has been performed twice. (B) Graph of transgenic *wmk* expression compared to *Drosophila* housekeeping gene *rp49*. Each point (n = 6) represents a pool of 30 embryos from a set of 10 mothers and 2 fathers. Values denote 2^-ΔCt^. Statistics are based on a Mann-Whitney U test. This experiment has been performed once. **p<0.01, ***p<0.001.
(TIF)

S4 Fig The *wmk* phenotype is not due to other forms of reproductive parasitism and it does not induce or rescue CI. (A) Resulting offspring sex ratios from infected mothers are shown here. Sex ratios of infected offspring of the indicated genotypes demonstrate that an infected background does not rescue or alter the *Act5c* driver-induced phenotype, which would be a characteristic of CI. Each point represents the offspring of a single vial of mothers and fathers. Average N per vial is 105 offspring. The group labeled “a” is significantly different compared to groups labeled “b”. Bars represent the average sex ratio. E = expressing, NE = non-expressing, *Act5c* = has *Act5c* gene, CyO = has CyO chromosome. (B) Hatch rate of offspring with parents expressing *wmk* under a *nanos* driver (expression in the gonads) in either fathers or mothers to test CI induction or rescue, respectively. Expression in males does not recapitulate wild type (WT) CI, and expression in females does not recapitulate rescue. Each dot represents the hatch rate of offspring of a single male and female, N = 24–36 crosses per group. Bars indicate average ± s. d. (C) Same as (B), but offspring have parents dually expressing *wmk*; WD0625 under a *nanos* driver (expression in gonads) in either fathers or mothers to test CI induction or rescue, respectively. Dual expression in males does not recapitulate WT CI, and dual expression in females does not recapitulate rescue CI. (D) Ratio of expressing to non-expressing flies (same flies as Fig 2) broken down by sex (ie, expressing males compared to non-expressing males, expressing females compared to non-expressing females). Each dot represents a comparison of sibling (brothers or sisters) offspring from a single vial of mothers and fathers. Bars represent the average ratio. The *wmk* male ratio is reduced, but *wmk* females are not significantly increased compared to controls. This indicates a loss of *wmk*-expressing males without a corresponding increase in females, suggesting male killing rather than feminization. Statistics for (A) and (D) experiments are based on a Kruskal-Wallis one-way ANOVA followed by Dunn’s correction. Statistics for (B) and (C) were performed with a Mann-Whitney U test between each of the two groups indicated by the significance bars. These experiments have all been performed once. *p<0.05, **p<0.01, ***p<0.001. (-), no *Wolbachia* infection; (+), *Wolbachia* infection; blue, normal; red, CI cross; purple, rescue cross; orange, *wmk* cross; green, dual *wmk*; WD0625.
(TIF)

S5 Fig The corresponding sex ratios of all experiments are female-biased. Alongside the experiments in Figs 3 and 4, sex ratios were measured. (A) Graph of the adult offspring sex ratio from the cytology experiment in Fig 3. Each point represents the offspring of a single vial of mothers and fathers. This was measured with offspring of siblings to the flies used to lay eggs in Fig 3. Average N is 79 adult offspring per cross of 10 mothers and 2 fathers. Statistics are based on a Kruskal-Wallis one-way ANOVA followed by Dunn’s correction. This experiment has been done once. (B) Graph of the adult sex ratio from the experiment in Fig 4. Each point represents the offspring of a single vial of mothers and fathers. This was measured with offspring of siblings to the flies used to lay eggs in Fig 4. Average N is 85 adult offspring per vial. Statistics are based on a Kruskal-Wallis one-way ANOVA followed by Dunn’s correction. E = expressing, NE = non-expressing, *Act5c* = has *Act5c* gene, CyO = has CyO chromosome. *p<0.05, **p<0.01, ***p<0.001. This experiment has been done once. All bars represent the average sex ratio.
(TIF)

S6 Fig Representative images of FISH staining of Y chromosome from data in Fig 3. These images were taken as a part of the experiment described in Fig 4. (A) Image of two normal control gene embryos approximately 4 h after egg deposition (AED) stained for DNA with PI. (B) Image of the same embryos as (A) stained with a Cy5-conjugated FISH probe specific to the Y chromosome. The left embryo is male, the right embryo is female. (C) Image of a *wmk* embryo 3–4 h AED stained with PI showing local mitotic failure and chromatin bridging. (D) Image of the same embryo as (C) stained with the Y chromosome probe, showing it is male.
(TIF)

S7 Fig Control gene females do not show DNA damage. These images were taken as a part of the experiment described in Fig 5, and all three are of the same embryo. (A) Image of a normal control gene female stained with DAPI for DNA at 4–5 h AED. (B) Image of the embryo stained with an antibody for pH2Av, demonstrating only background signal. (C) Image of the embryo stained with an antibody for H4K16ac, demonstrating no detectable signal.
(TIF)

S8 Fig Predicted protein architecture of Wmk homologs and homology to a phage repressor. (A) Image of the most likely 3D structure of Wmk from *w*Mel determined by the Phyre2 web portal. 66% of residues modeled at 99.9% confidence. (B) The known protein structure with the most shared similarity across all homologs, with the highest sequence identity and confidence, is a known phage DNA-binding transcriptional repressor. Namely, it is the Rep-Ant complex from *Salmonella*-temperate phage, modeled here (99.8% confidence and 19% residue identity compared to *w*Mel Wmk). Other top results were also almost exclusively transcriptional regulators and DNA-binding proteins. The Rep-Ant complex is comprised of two separate, dimerized peptides, and does not include the linker region of Wmk shown in green in (A). (C) Image of the most likely 3D structure of Wmk from *w*Bif determined by Phyre2. 73% of residues modeled at 99.9% confidence. (D) Image of the most likely 3D structure of Wmk from *w*Inn/*w*Bor (same sequence) determined by Phyre2. 59% of residues modeled at 99.9% confidence. (E) Image of the most likely 3D structure of Wmk from *w*Rec determined by Phyre2. 62% of residues modeled at 99.9% confidence. All images are colored in order of the rainbow from N terminus (red) to C terminus (blue). Although there are no breaks in input sequence, some breaks are shown in the models because of low confidence in modeling in those regions (the linker region between the two alpha helix bundles).
(TIF)

S9 Fig Wmk amino acid identity is more conserved in the DNA-binding domains than certain other regions of the protein. (A) Level of amino acid conservation is shown across the length of the amino acid alignment of 31 Wmk homologs. The homologs used in the analysis include all those shown in S1 Fig with the addition of the *w*Bif homolog. A score of 1 indicates complete conservation across homologs while a score of 0 indicates all homologs have different amino acid identities in that location. The two HTH DNA-binding domains are highlighted in shades of orange for reference. (B) Amino acid conservation from the same set of data as (A) is shown in a different format here, where each dot represents the conservation of a particular amino acid position within a designated region of the protein. Statistics are based on a Kruskal-Wallis one-way ANOVA followed by Dunn’s correction. Bars indicate mean values. Colors of HTH regions correspond to (A) and shades of blue are used to distinguish other regions. **p<0.01, ***p<0.001, ****p<0.0001.
(TIF)

S1 Table Full details of comparative genomics analysis for male-killing gene candidates. All *w*Bif phage genes are listed with scaffold numbers and annotations. Mobile elements were removed from further analysis, but all other genes were evaluated for presence in other strains, disruptions in other strains, E^-20^ thresholds. Remaining genes that fit all criteria were included in the final candidate list (Table 1).
(XLSX)

S2 Table Homologs of Wmk from related bacterial strains. All non-*w*Mel *Wolbachia* Wmk homologs with a reciprocal BLASTp E-value of E^-15^ or above were included, and all have a reciprocal best BLAST (RBB) of the Wmk protein in *w*Mel. Accession numbers for NCBI are included.
(XLSX)

S3 Table Primers used in this study. All primers, names, and sequences are listed, along with the Y-chromosome FISH probe sequence that also has a 5’ Cy5 tag.
(XLSX)

S4 Table Wmk protein homologs included in S1B Fig. All homologs listed are those used for S1B Fig. Accession numbers for NCBI are included.
(XLSX)

S5 Table *wmk* gene homologs included in S1A Fig. All homologs listed are those that were included in the phylogeny from S1A Fig., or were excluded for indicated reasons. Accession numbers for NCBI are included.
(XLSX)

S6 Table Amino acid similarity between Wmk and homologs in male-killing strains. Strains include all those that are currently sequenced.
(XLSX)
