# Supplementary figures and images for: The phage gene wmk is a candidate for male killing by a bacterial endosymbiont
Source: PLoS Pathog. 2019 Sep 10;15(9):e1007936. doi: 10.1371/journal.ppat.1007936 (PMC6736233; doi:10.1371/journal.ppat.1007936)

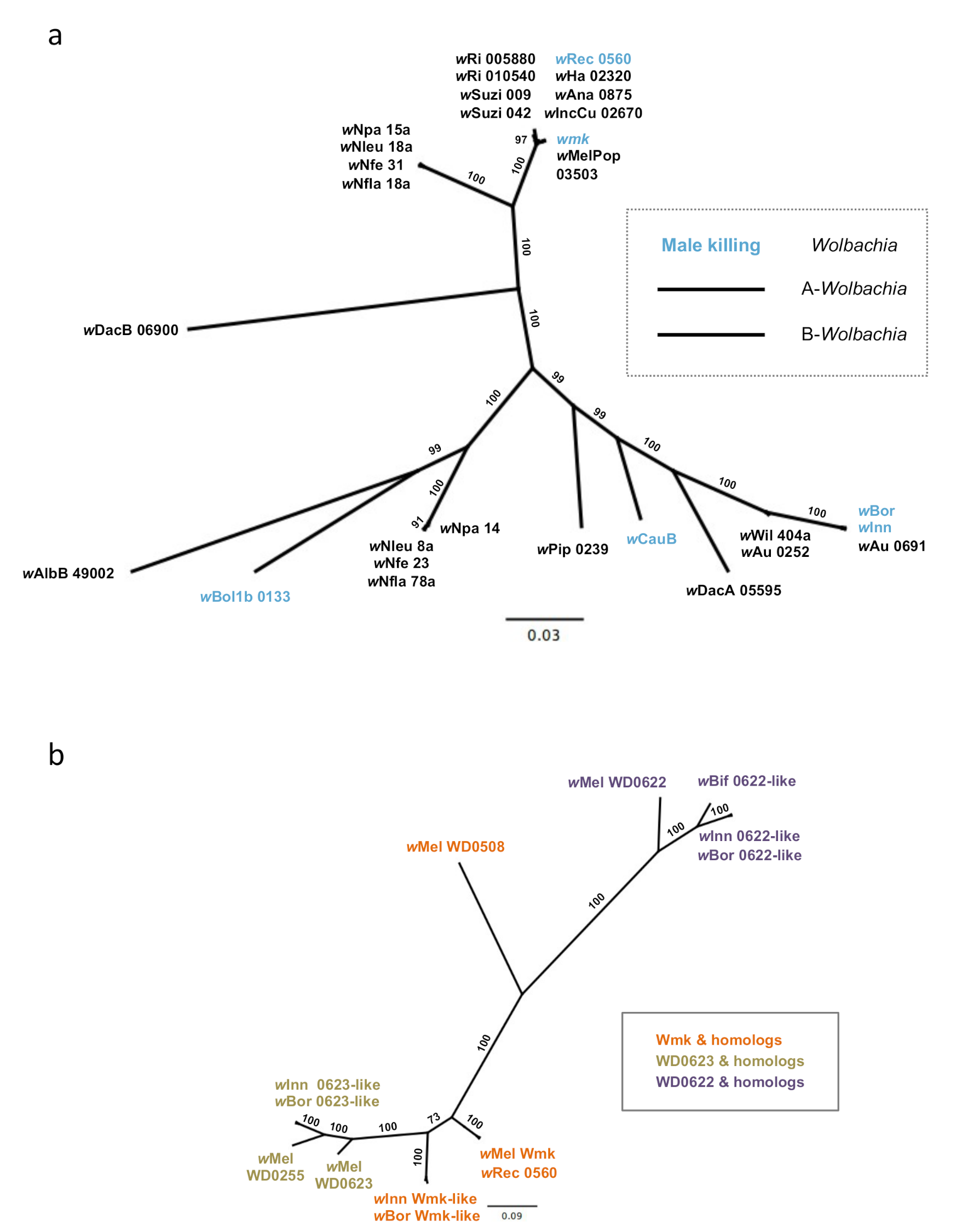

Supplement: S1 Fig — (A) Phylogeny of full-length wmk gene, based on an 893-bp alignment and GTR+I+G model of evolution. Full-length wmk homologs are widespread throughout prophage WO-containing Wolbachia strains, some of which are male-killing strains. Like many WO-associated genes, including CI factors cifA and cifB, the wmk phylogeny does not support evolution with the Wolbachia chromosome because homologs in A- and B-Wolbachia do not cluster according to supergroup. Wolbachia supergroups are illustrated as either black (A-Wolbachia) or red (B-Wolbachia) branches. wmk (WD0626) and homologs from male-killing strains are highlighted in cyan. Consensus support values are shown on the branches. The tree includes all taxa that are reciprocal best hits of wMel wmk. See S5 Table for accession numbers and BLASTn E-values. (B) A Bayesian phylogeny of Wmk protein and homologous peptides from wMel and sequenced male-killing strains in Drosophila, based on a 168 aa-alignment using the JTT+G model of evolution. It shows that homologs in these taxa cluster according to gene synteny within prophage WO genomes (see Fig 1). Consensus support values are shown on the branches. Colors correspond to Fig 1. Accession numbers and BLASTP E-values are provided in S4 Table. (TIF) [file ppat.1007936.s001.tif]

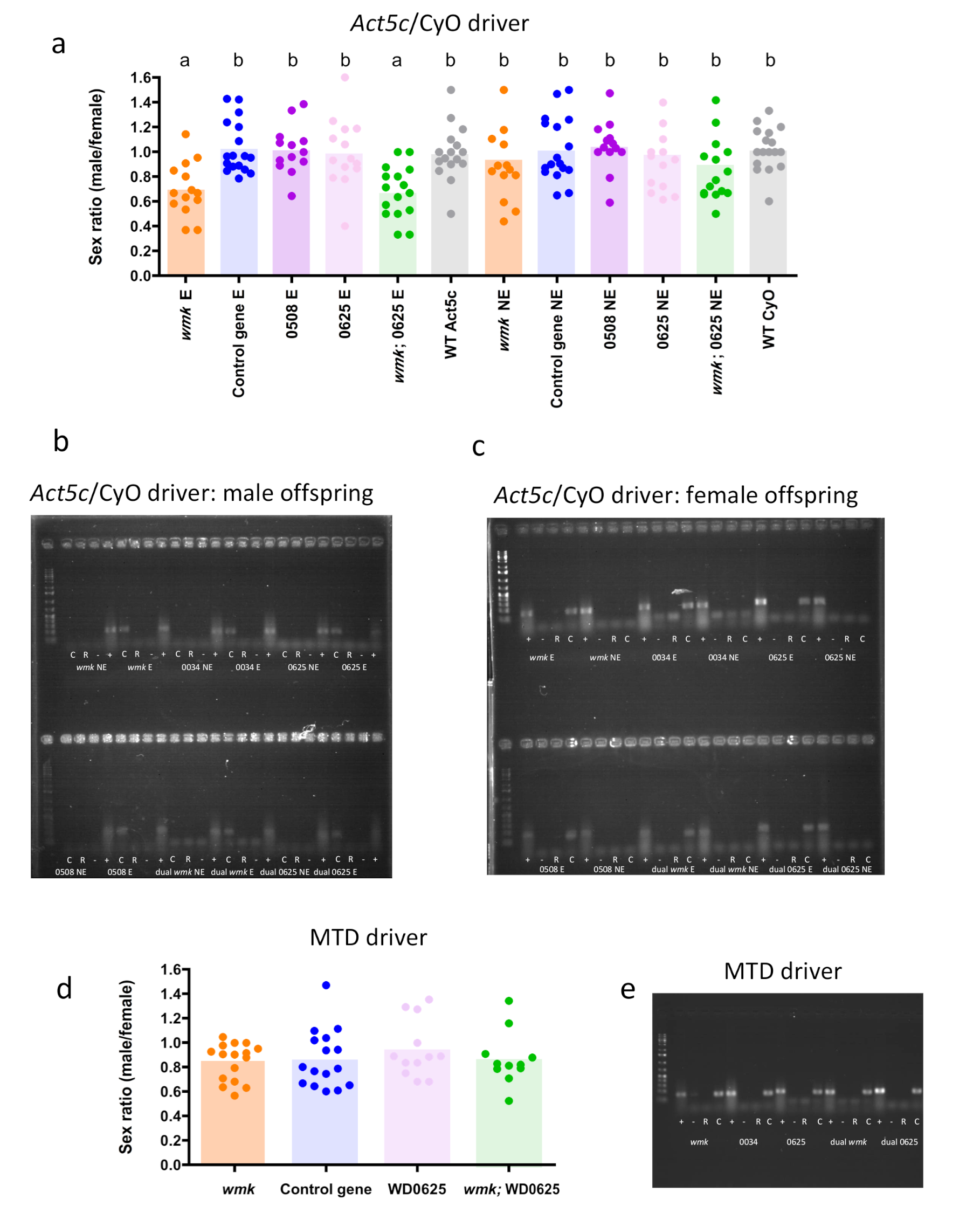

Supplement: S2 Fig — (A) Sex ratios were quantified for wmk, the control gene (WD0034), WD0625, WD0508, or dual wmk;WD0625 transgenes expressed with the Act5c/CyO driver. Expressing and non-expressing flies of each genotype are siblings. Each point represents the offspring of one vial of 10 mothers and 2 fathers. A biased sex ratio only results when wmk is expressed. Average N per vial is 78. Statistics are based on a Kruskal-Wallis one-way ANOVA followed by Dunn’s correction with only the non-expressing flies or only the expressing flies. Groups labeled “a” are significantly different compared to groups labeled “b”. Non-expressing flies are non-significant. Bars represent the average sex ratio. E = expressing, NE = non-expressing, Act5c = Act5c gene present, CyO = CyO chromosome present. This experiment has been performed once. (B) Transgenes are expressed in Act5c (E) adult males but not their CyO (NE) brothers as demonstrated by cDNA generated from males. Samples were taken from offspring of parental siblings from the experiment in (A). Samples were from pooled, whole-body, adult extractions of three individuals from each genotype. (C) Transgenes are expressed in Act5c (E) females, but not their CyO (NE) sisters as demonstrated by cDNA generated from females. See (B) for details. Both (B) and (C) have been performed once. (D) Sex ratios were similarly quantified for the listed transgenes using a maternal triple driver (MTD) where expression was driven in the mother throughout oogenesis and offspring were laid with the expressed products loaded into the eggs. Each point represents the offspring of a vial of 10 mothers and 2 fathers. Average N per vial is 74. Statistics are based on a Kruskal-Wallis one-way ANOVA followed by Dunn’s correction. Bars represent the average sex ratio. This experiment has been done once. (E) Transgenes are expressed in all adult offspring from the MTD driver as demonstrated by cDNA generated from siblings of mothers from the experiment in (D). Samples are f [file ppat.1007936.s002.tif]

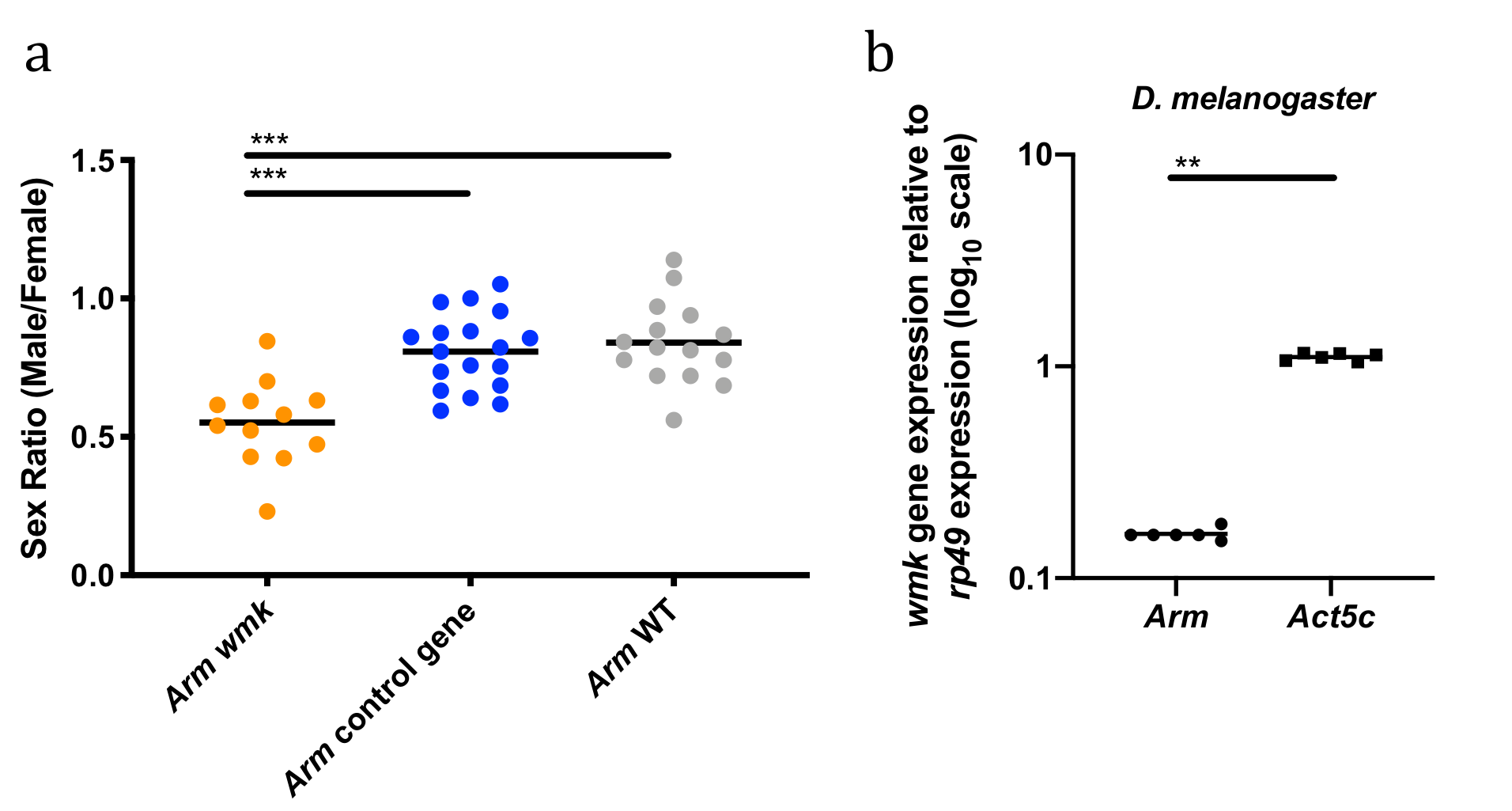

Supplement: S3 Fig — (A) Sex ratios were quantified for wmk, the control gene (WD0034), and WT flies. The armadillo (arm) driver is homozygous, so all offspring express the gene. Each point represents the offspring of one vial of 10 mothers and 2 fathers. A biased sex ratio results only when wmk is expressed, as with Act5c (Fig 2). Average N per vial is 73. Statistics are based on a Kruskal-Wallis one-way ANOVA followed by Dunn’s correction. This experiment has been performed twice. (B) Graph of transgenic wmk expression compared to Drosophila housekeeping gene rp49. Each point (n = 6) represents a pool of 30 embryos from a set of 10 mothers and 2 fathers. Values denote 2-ΔCt. Statistics are based on a Mann-Whitney U test. This experiment has been performed once. **p<0.01, ***p<0.001. (TIF) [file ppat.1007936.s003.tif]

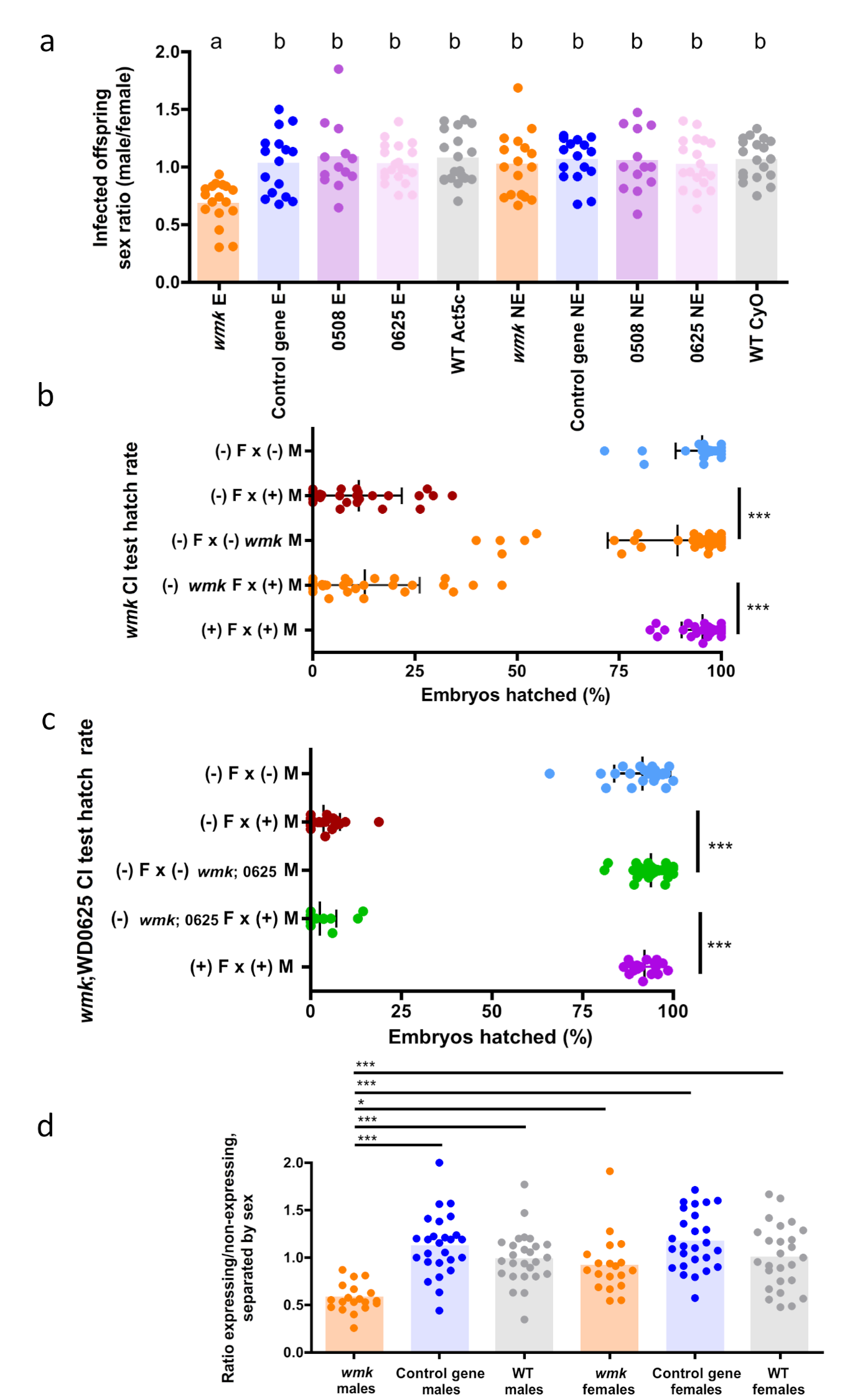

Supplement: S4 Fig — (A) Resulting offspring sex ratios from infected mothers are shown here. Sex ratios of infected offspring of the indicated genotypes demonstrate that an infected background does not rescue or alter the Act5c driver-induced phenotype, which would be a characteristic of CI. Each point represents the offspring of a single vial of mothers and fathers. Average N per vial is 105 offspring. The group labeled “a” is significantly different compared to groups labeled “b”. Bars represent the average sex ratio. E = expressing, NE = non-expressing, Act5c = has Act5c gene, CyO = has CyO chromosome. (B) Hatch rate of offspring with parents expressing wmk under a nanos driver (expression in the gonads) in either fathers or mothers to test CI induction or rescue, respectively. Expression in males does not recapitulate wild type (WT) CI, and expression in females does not recapitulate rescue. Each dot represents the hatch rate of offspring of a single male and female, N = 24–36 crosses per group. Bars indicate average ± s. d. (C) Same as (B), but offspring have parents dually expressing wmk; WD0625 under a nanos driver (expression in gonads) in either fathers or mothers to test CI induction or rescue, respectively. Dual expression in males does not recapitulate WT CI, and dual expression in females does not recapitulate rescue CI. (D) Ratio of expressing to non-expressing flies (same flies as Fig 2) broken down by sex (ie, expressing males compared to non-expressing males, expressing females compared to non-expressing females). Each dot represents a comparison of sibling (brothers or sisters) offspring from a single vial of mothers and fathers. Bars represent the average ratio. The wmk male ratio is reduced, but wmk females are not significantly increased compared to controls. This indicates a loss of wmk-expressing males without a corresponding increase in females, suggesting male killing rather than feminization. Statistics for (A) and (D) experiments are based on a Kruskal-Wallis [file ppat.1007936.s004.tif]

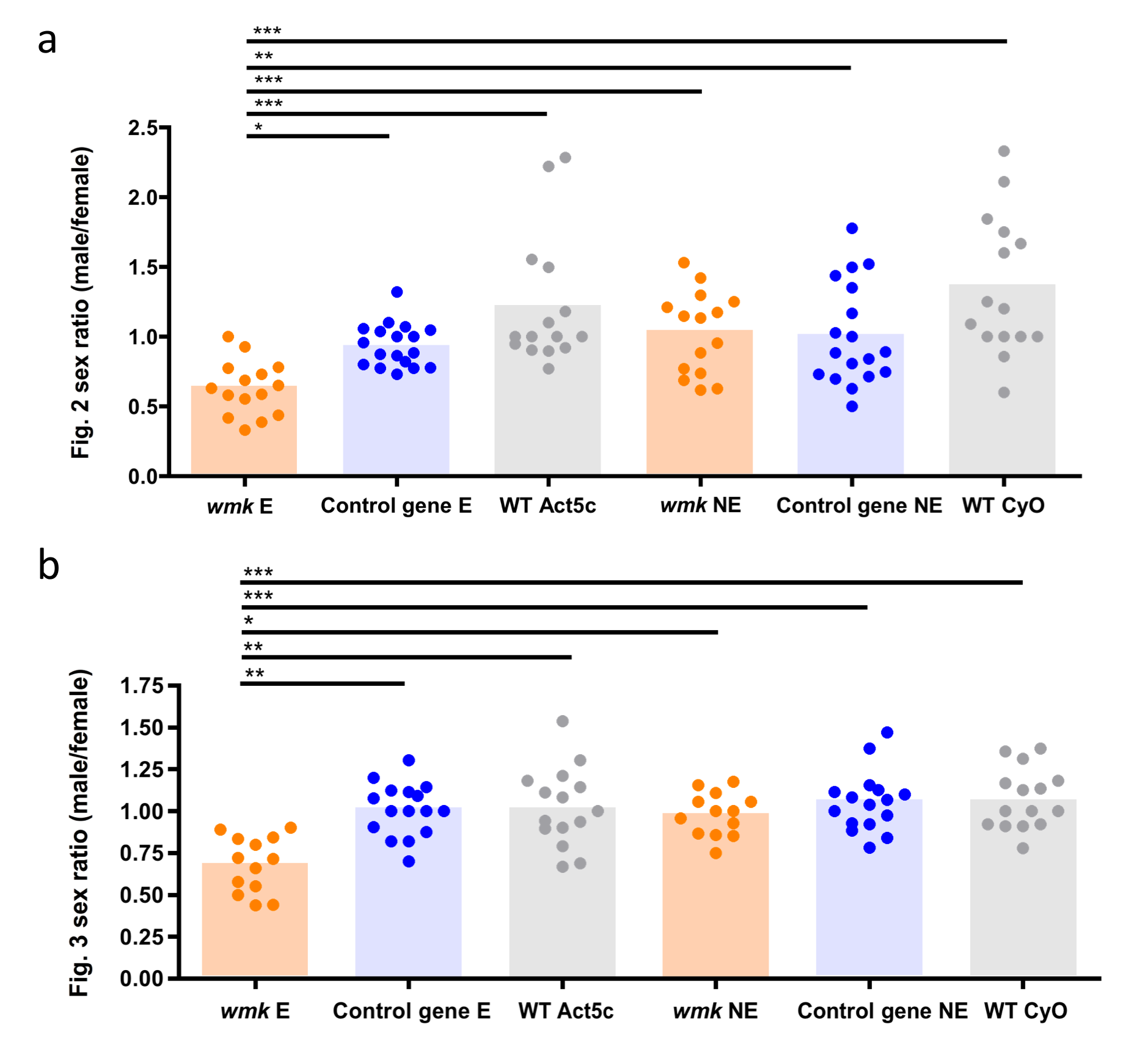

Supplement: S5 Fig — Alongside the experiments in Figs 3 and 4, sex ratios were measured. (A) Graph of the adult offspring sex ratio from the cytology experiment in Fig 3. Each point represents the offspring of a single vial of mothers and fathers. This was measured with offspring of siblings to the flies used to lay eggs in Fig 3. Average N is 79 adult offspring per cross of 10 mothers and 2 fathers. Statistics are based on a Kruskal-Wallis one-way ANOVA followed by Dunn’s correction. This experiment has been done once. (B) Graph of the adult sex ratio from the experiment in Fig 4. Each point represents the offspring of a single vial of mothers and fathers. This was measured with offspring of siblings to the flies used to lay eggs in Fig 4. Average N is 85 adult offspring per vial. Statistics are based on a Kruskal-Wallis one-way ANOVA followed by Dunn’s correction. E = expressing, NE = non-expressing, Act5c = has Act5c gene, CyO = has CyO chromosome. *p<0.05, **p<0.01, ***p<0.001. This experiment has been done once. All bars represent the average sex ratio. (TIF) [file ppat.1007936.s005.tif]

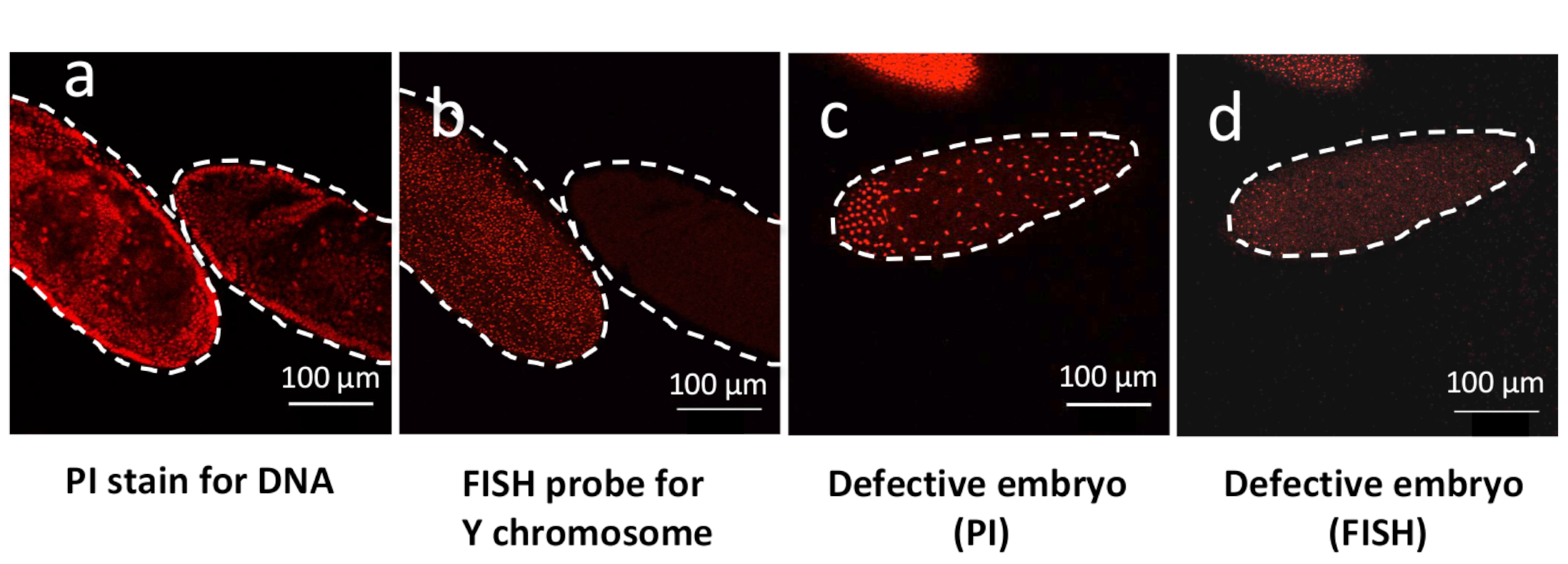

Supplement: S6 Fig — These images were taken as a part of the experiment described in Fig 4. (A) Image of two normal control gene embryos approximately 4 h after egg deposition (AED) stained for DNA with PI. (B) Image of the same embryos as (A) stained with a Cy5-conjugated FISH probe specific to the Y chromosome. The left embryo is male, the right embryo is female. (C) Image of a wmk embryo 3–4 h AED stained with PI showing local mitotic failure and chromatin bridging. (D) Image of the same embryo as (C) stained with the Y chromosome probe, showing it is male. (TIF) [file ppat.1007936.s006.tif]

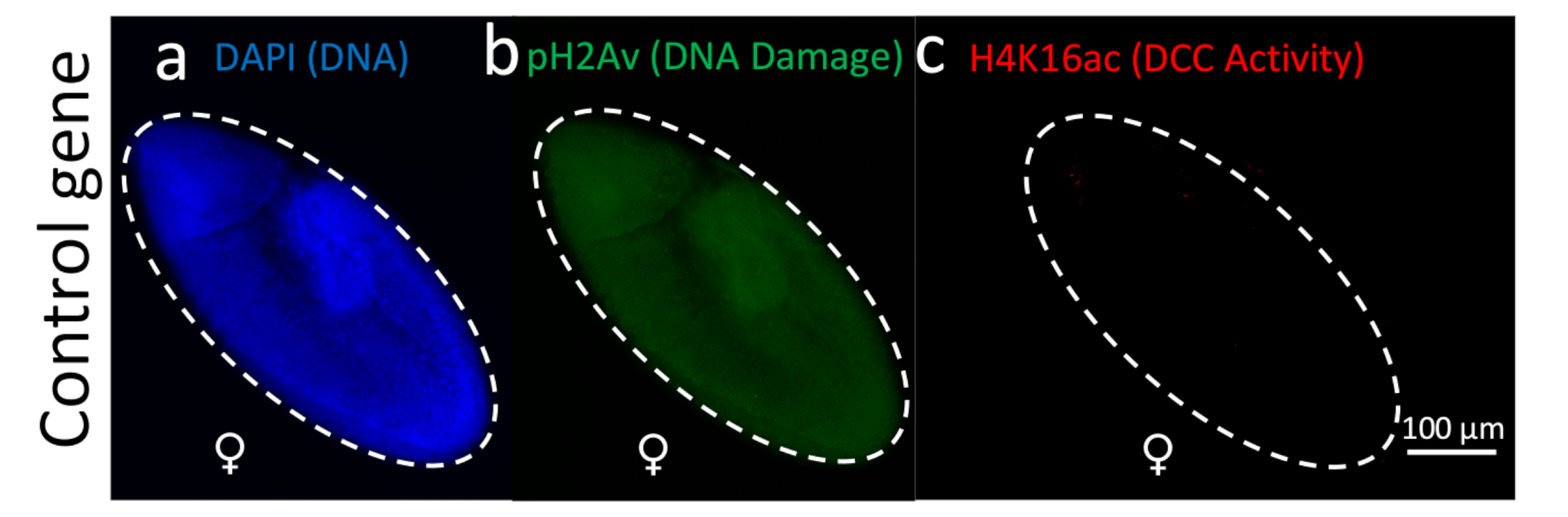

Supplement: S7 Fig — These images were taken as a part of the experiment described in Fig 5, and all three are of the same embryo. (A) Image of a normal control gene female stained with DAPI for DNA at 4–5 h AED. (B) Image of the embryo stained with an antibody for pH2Av, demonstrating only background signal. (C) Image of the embryo stained with an antibody for H4K16ac, demonstrating no detectable signal. (TIF) [file ppat.1007936.s007.tif]

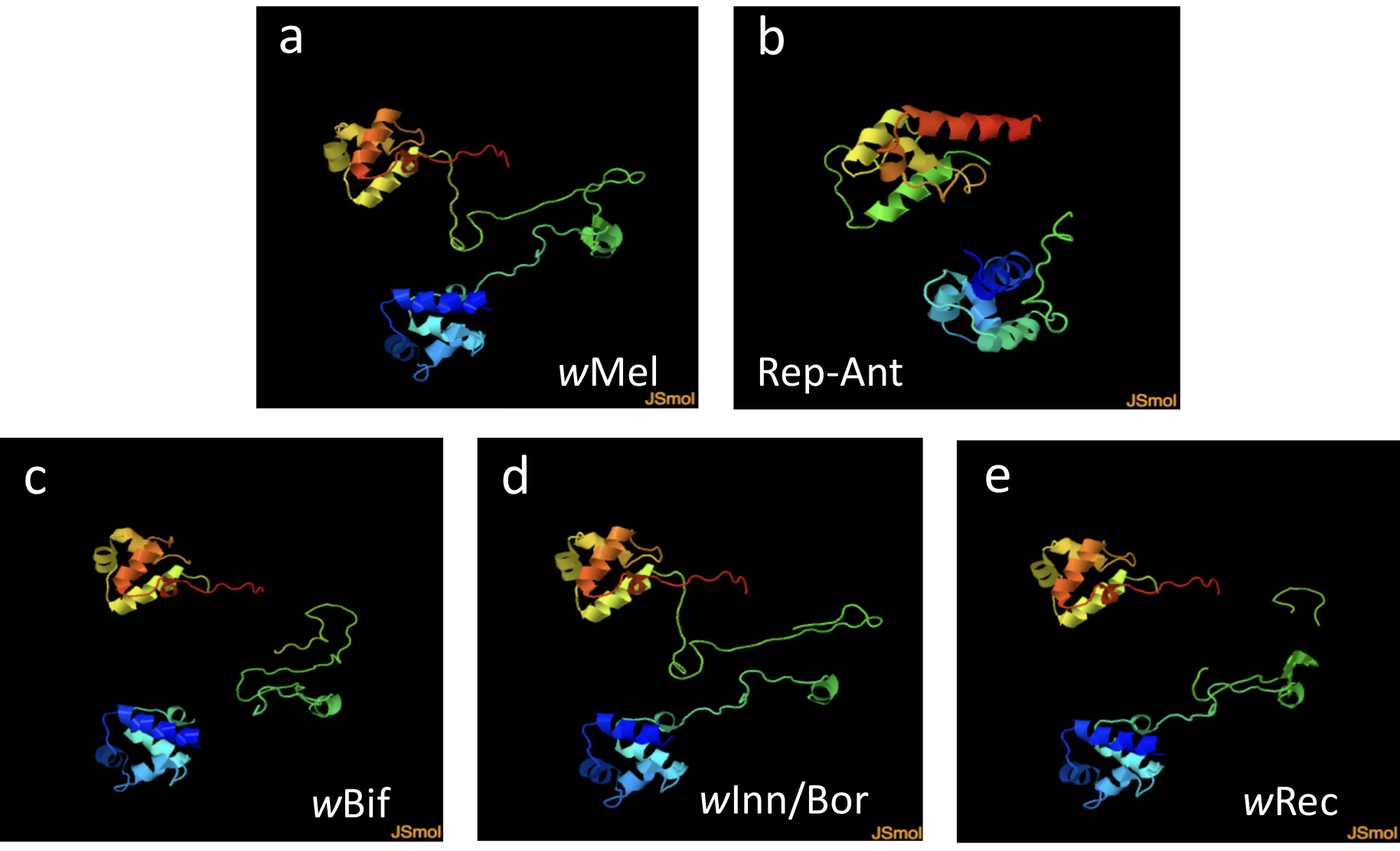

Supplement: S8 Fig — (A) Image of the most likely 3D structure of Wmk from wMel determined by the Phyre2 web portal. 66% of residues modeled at 99.9% confidence. (B) The known protein structure with the most shared similarity across all homologs, with the highest sequence identity and confidence, is a known phage DNA-binding transcriptional repressor. Namely, it is the Rep-Ant complex from Salmonella-temperate phage, modeled here (99.8% confidence and 19% residue identity compared to wMel Wmk). Other top results were also almost exclusively transcriptional regulators and DNA-binding proteins. The Rep-Ant complex is comprised of two separate, dimerized peptides, and does not include the linker region of Wmk shown in green in (A). (C) Image of the most likely 3D structure of Wmk from wBif determined by Phyre2. 73% of residues modeled at 99.9% confidence. (D) Image of the most likely 3D structure of Wmk from wInn/wBor (same sequence) determined by Phyre2. 59% of residues modeled at 99.9% confidence. (E) Image of the most likely 3D structure of Wmk from wRec determined by Phyre2. 62% of residues modeled at 99.9% confidence. All images are colored in order of the rainbow from N terminus (red) to C terminus (blue). Although there are no breaks in input sequence, some breaks are shown in the models because of low confidence in modeling in those regions (the linker region between the two alpha helix bundles). (TIF) [file ppat.1007936.s008.tif]

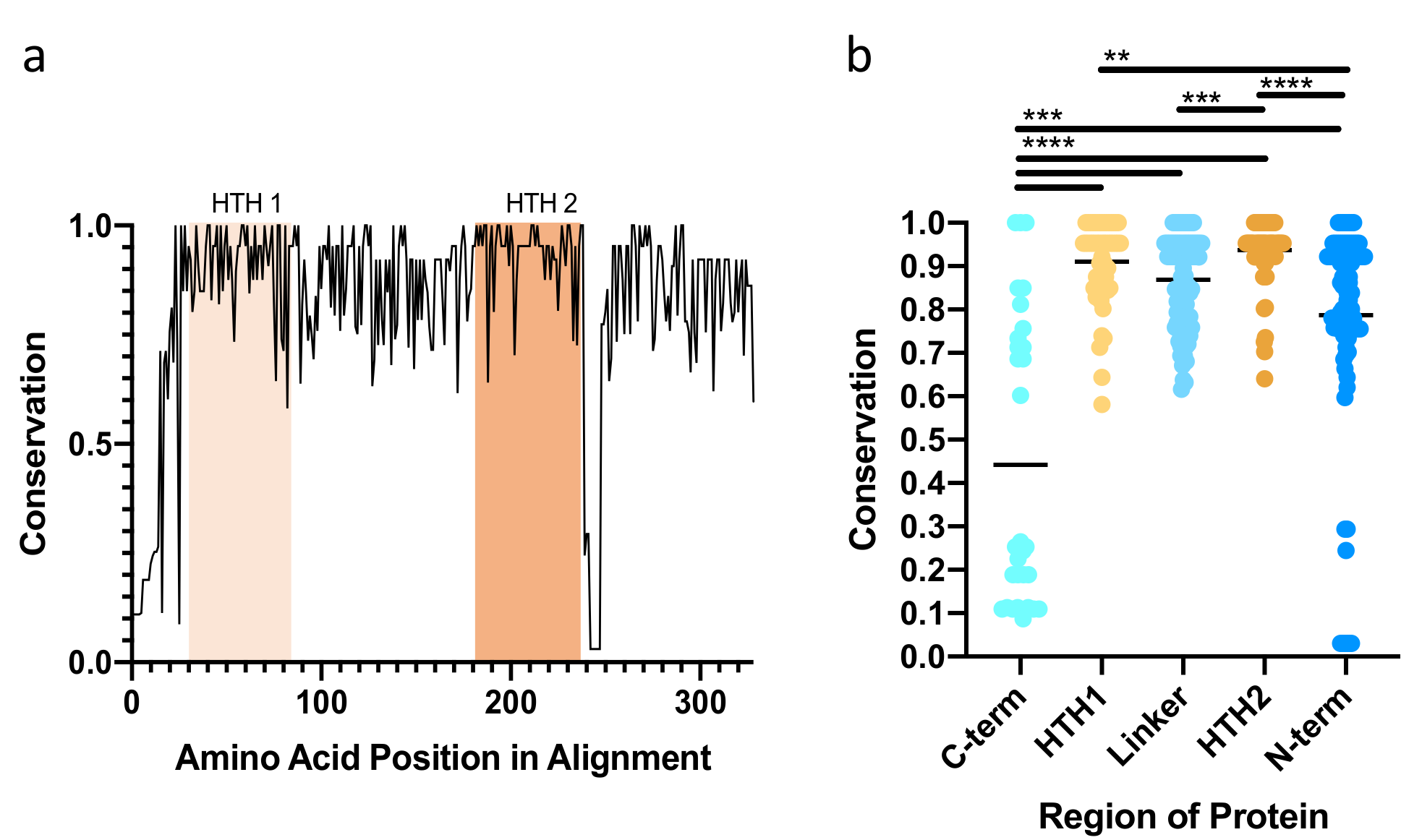

Supplement: S9 Fig — (A) Level of amino acid conservation is shown across the length of the amino acid alignment of 31 Wmk homologs. The homologs used in the analysis include all those shown in S1 Fig with the addition of the wBif homolog. A score of 1 indicates complete conservation across homologs while a score of 0 indicates all homologs have different amino acid identities in that location. The two HTH DNA-binding domains are highlighted in shades of orange for reference. (B) Amino acid conservation from the same set of data as (A) is shown in a different format here, where each dot represents the conservation of a particular amino acid position within a designated region of the protein. Statistics are based on a Kruskal-Wallis one-way ANOVA followed by Dunn’s correction. Bars indicate mean values. Colors of HTH regions correspond to (A) and shades of blue are used to distinguish other regions. **p<0.01, ***p<0.001, ****p<0.0001. (TIF) [file ppat.1007936.s009.tif]
